# An innovative non-invasive technique for subcutaneous tumour measurements

**DOI:** 10.1371/journal.pone.0216690

**Published:** 2019-10-14

**Authors:** Juan Delgado-SanMartin, Beate Ehrhardt, Marcin Paczkowski, Sean Hackett, Andrew Smith, Wajahat Waraich, James Klatzow, Adeala Zabair, Anna Chabokdast, Leonardo Rubio-Navarro, Amar Rahi, Zena Wilson

**Affiliations:** 1 Fuel3D, Oxford Science Park, Oxford, England, United Kingdom; 2 AstraZeneca IMED Biotech Unit, Discovery Sciences, Cambridge Science Park, Cambridge, England, United Kingdom; 3 AstraZeneca IMED Biotech Unit, Oncology, Alderley Park, Macclesfield, England, United Kingdom; Stanford University, UNITED STATES

## Abstract

**Introduction:**

In oncological drug development, animal studies continue to play a central role in which the volume of subcutaneous tumours is monitored to assess the efficacy of new drugs. The tumour volume is estimated by taking the volume to be that of a regular spheroid with the same dimensions. However, this method is subjective, insufficiently traceable, and is subject to error in the accuracy of volume estimates as tumours are frequently irregular.

**Methods & results:**

This paper reviews the standard technique for tumour volume assessment, calliper measurements, by conducting a statistical review of a large dataset consisting of 2,500 tumour volume measurements from 1,600 mice by multiple operators across 6 mouse strains and 20 tumour models. Additionally, we explore the impact of six different tumour morphologies on volume estimation and the detection of treatment effects using a computational tumour growth model. Finally, we propose an alternative method to callipers for estimating volume–BioVolume^TM^, a 3D scanning technique. BioVolume simultaneously captures both stereo RGB (Red, Green and Blue) images from different light sources and infrared thermal images of the tumour in under a second. It then detects the tumour region automatically and estimates the tumour volume in under a minute. Furthermore, images can be processed in parallel within the cloud and so the time required to process multiple images is similar to that required for a single image. We present data of a pre-production unit test consisting of 297 scans from over 120 mice collected by four different operators.

**Conclusion:**

This work demonstrates that it is possible to record tumour measurements in a rapid minimally invasive, morphology-independent way, and with less human-bias compared to callipers, whilst also improving data traceability. Furthermore, the images collected by BioVolume may be useful, for example, as a source of biomarkers for animal welfare and secondary drug toxicity / efficacy.

## Introduction

Animal models of human cancers are fundamental to our understanding of tumour biology. Tumour volume is a significant metric for preclinical trials where it provides a surrogate measure of both disease progression and treatment efficacy. Thus, accurate and repeatable estimation of tumour volume is crucial to declare a given trial to be a success or failure with confidence [1]. At present, it is standard practice to estimate subcutaneous tumour volume by using callipers to take manual measurements of tumour length and width. This approach assumes tumours to be regular spheroids [1,2], whereas tumours are often irregular. Medical imaging technologies such as magnetic resonance imaging (MRI), computed tomography (CT), and ultrasound (US) offer an alternative but require the immobilisation of the animals (often by anaesthesia) and are resource intensive, thereby potentially compromising animal welfare, increasing costs, and creating logistical complications [3–6]. Efforts have been made to produce more accessible alternatives to these methods, such as 3D stereo photometry, time-of-flight, and structured light [7–11].

In what follows, we explore how manual calliper measurements introduce human bias into pre-clinical trials. Furthermore, we use a cellular automaton model to investigate how calliper measurements influence our understanding of study outcomes for six different tumour morphologies [12]. We propose an alternative measurement method, named *BioVolume*^*TM*^ *(Fuel3D*, *Oxford*, *UK;* *www*.*fuel3d*.*com**)* which combines 3D stereo photometry (to capture depth) and infrared/thermal imaging (to delineate the poorly vascularised tumour region).

## Methods

### Datasets

We analysed two datasets:

- Dataset 1, Calliper statistical review: Records for 1,608 mice and 2,488 calliper measurements were collected by 29 AstraZeneca operators over a period of 17 months from February 2017 until June 2018. The measurements belong to 43 pre-clinical studies (S1 Dataset).
- Dataset 2, BioVolume–calliper comparison: We collected scans of tumours from 120 mice using a pre-production test unit of BioVolume on 3 occasions between 14/09/18 and 05/10/18. Calliper measurements were also taken for each tumour. A total of 257 calliper measurements and 297 scans were collected by four operators (S2 Dataset).

Five different strains of mice were sourced from Charles River UK (www.criver.com) & Envigo UK (www.envigo.com): SCID, BALB/c, C57BL/6, NSG and Nude. Hairy mice were shaven as usual for the experiments. More information regarding the data can be found in S1 Appendix.

### Biovolume^TM^

**Fig 1:**
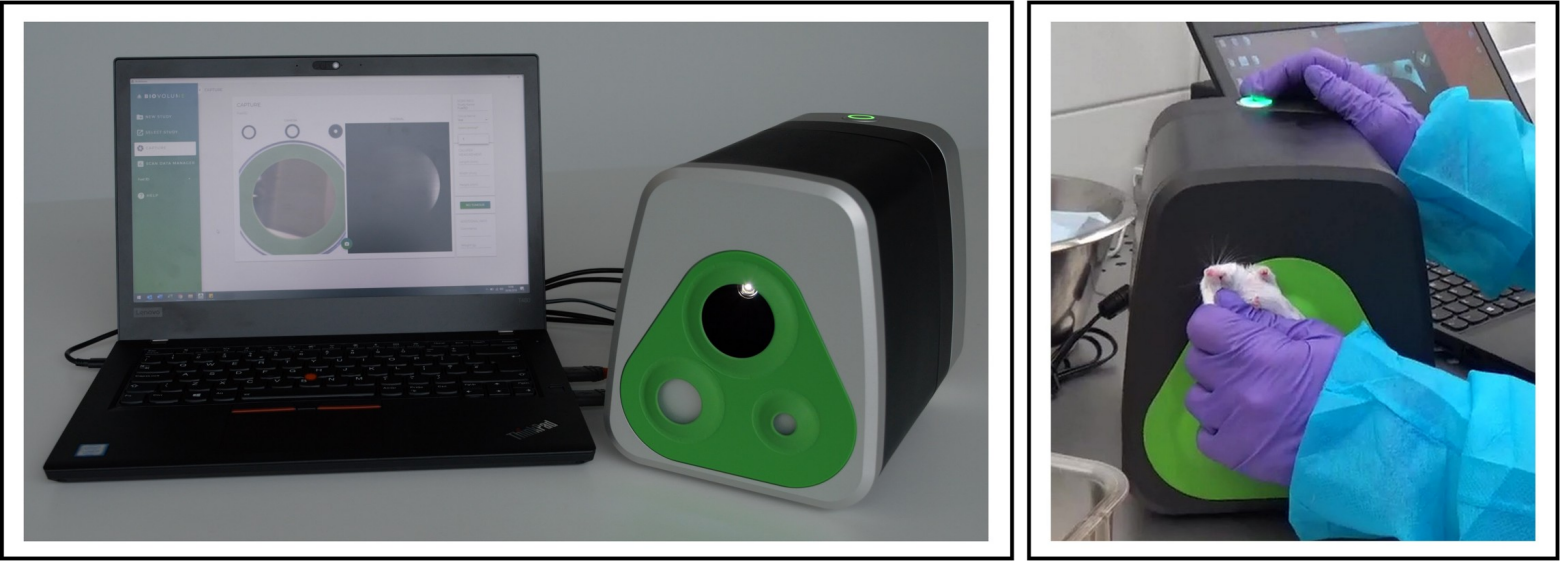
Experimental setup. Complete set up of the BioVolume unit including computer monitor and desktop device (left), closeup image of a white SCID mouse being presented to the aperture of BioVolume (right).

BioVolume is a small desktop device (27 x 18.5 x 16.8 cm), which captures both thermal (infrared) and 3D surface images. To acquire a scan, the shaven mouse is held such that the tumour region is exposed to the device aperture (see Fig 1, left). Then, acquisition is triggered on either a cable-connected laptop or by using a button on the device itself (see Fig 1). There is no requirement to anesthetise the animal. Acquisition takes around 0.25s, and rendering occurs in the cloud. Rendering a full image, segmentation, and measurement extraction requires approximately 25s depending on internet speed and these can be parallelised. The acquired measurements are then displayed to the operator immediately.

The BioVolume unit consists of a stereo system with two RGB cameras, three white light flashes, and an infrared thermal camera. Upon activation, the unit collects 6 photographic (RGB) images and a single thermal frame (see S1 Scan for a primary flash and thermal image example). The BioVolume software utilised in this work was a beta version named v0.1_f5bf15. The RGB images are reconstructed in a surface by means of a binocular stereo-process, outputting both depth and RGB maps [13,14]. The thermal frame is mapped onto the 3D reconstruction using a conventional affine transformation based on a prior positional calibration of the RGB and thermal cameras. The segmentation of the tumour happens on the thermal map, which is then projected onto the depth map. The height is then obtained by fitting a plane to the back of the mouse using an optimisation algorithm. More details are provided in S1 Appendix.

#### Volume calculation

We compare two formulae (1,2) for the estimation of tumour volume:

- Spheroid formula (BioVolume & callipers):
  $$V_{sph}=\frac{\pi }{6}\cdot length\cdot width^{2}$$ (1)
- Cylindrical volume (BioVolume):
  $$V_{cyl}=Area\cdot height$$ (2)

#### Computational tumour growth model

The cellular automaton model consisted of a rule-based model operating on two simulated cell populations growing on a 3D lattice. The rules and parametrisation originate from logical assumptions for tumour growth and treatment. There are four main parameters: vertical bias, cell division rate, magnitude, and length of treatment. These parameters are depicted in mathematical notation as: $\theta =\{bias,\;p_{divi},\lambda ,\lambda_{len}\}$ (see S2 Appendix). The model can produce six different morphologies (see results section below).

#### Data analysis

For the calliper data set, we focus on metrics for inter-operator repeatability and the consistency of BioVolume’s linear measurements with those of callipers. For the former, we use the coefficient of variation (CV) as a measure of precision and intra-class correlation (ICC) as a measure of reliability. The ICC provides a measure of the correlation between two different people acquiring scans from BioVolume on different occasions [15]. The coefficient of variation is found as $CV=\frac{\sigma }{\mu }$, where *σ* and *μ* are the standard deviation and mean, respectively (15). To assess the consistency between the linear measurements of both methods, we compared the length and width measurements of BioVolume and callipers using a two-sample one-sided t-test with unequal variance, adjusted for multiple testing [16]. For a limited number of cases, we were able to compare the calliper derived volumes to excised tumour weight. For these comparisons, we assumed the density of the tumours to be approximately that of most human soft tissues (0.90 (fat) -1.09 (skin) g/cm^3^) [17]. For the evaluation of the control (*V*_*c*_) and treated (*V*_*T*_) growth curves we used Tumour Growth Inhibition (TGI):

$$TGI=\left(1-\frac{V_{T}\left(t\right)\;V_{C}\left(0\right)}{V_{T}\left(0\right)\;V_{C}\left(t\right)}\right)\times 100\%,$$ (3)

and Area-Under-the-Curve (AUC) index:

$$AUC=\left(1-\frac{AUC_{T}}{AUC_{C}}\right)\times 100\%,$$ (4)

where “V” denotes volume, “t” is any given time after the beginning of the study where t = 0, *AUC*_*T*_,*AUC*_*C*_ area-under-the-curve for treated and control (see S2 Appendix).

A full description of the methods for the analysis of the calliper and BioVolume data can be found in S1 Appendix.

## Results

### Calliper statistical review

#### Inter-operator repeatability

**Fig 2:**
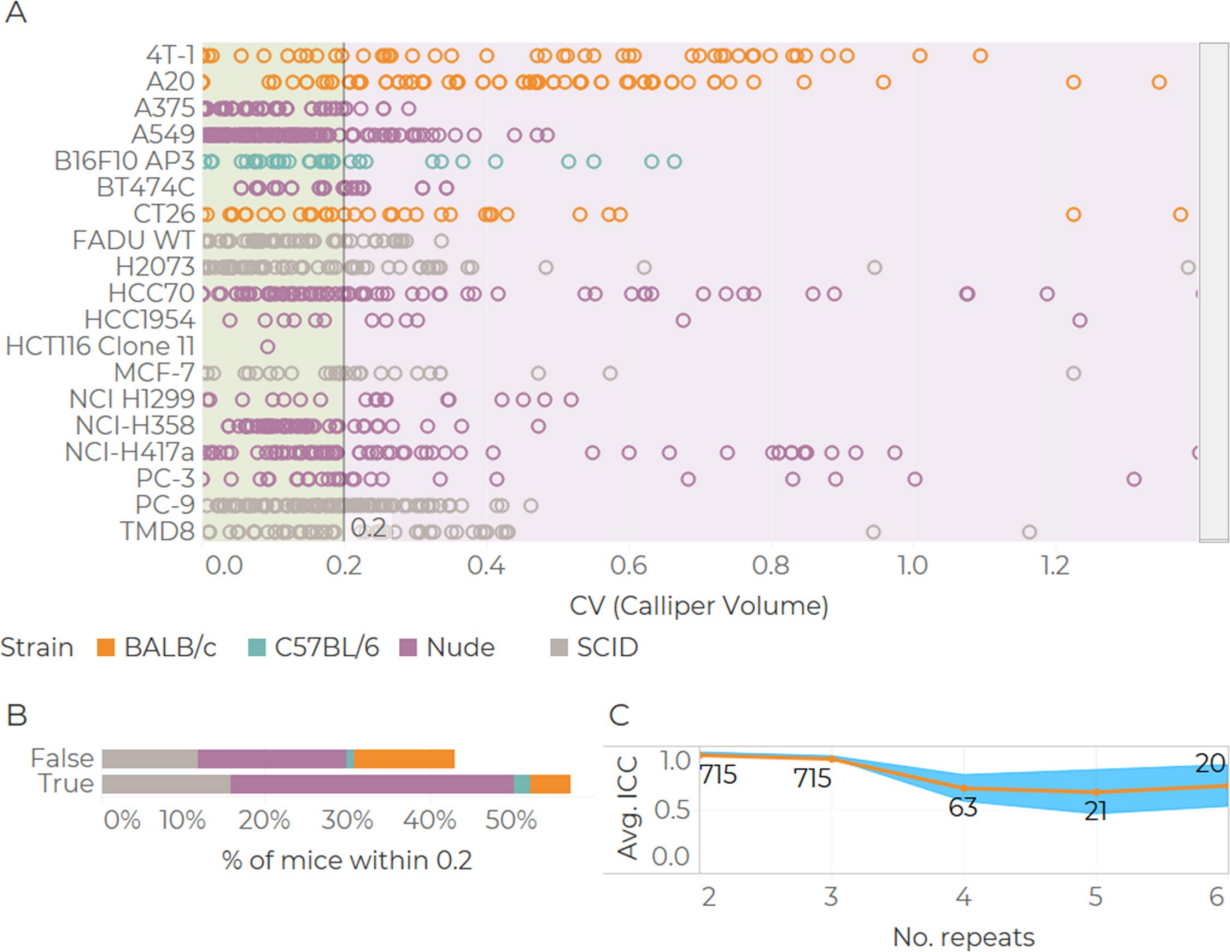
Summary of inter-operator precision and ICC in volume from callipers. Precision single values ordered by tumour model and mouse strain (A). Quantification of values within a precision limit of 0.2 (B). ICC values vs number of operators (C). Values printed on the plot indicate number of observations, dots are average ICC and shaded bars are 95% confidence intervals.

Inter-operator repeatability is a significant challenge when measuring subcutaneous tumours (see Introduction). It is of paramount importance that consistent and reliable measurements are made. For our evaluations we considered a Coefficient of Variation (CV) of less than 0.2 acceptable [15]. Fig 2A displays the inter-operator precision for each animal model and tumour cell line. Precision was lowest for cell lines 4T-1 and A20. Across all four mouse strains for which inter-operator metrics were attainable, the distribution of precision was comparable. 59.3% of the 968 points scored precision values below 0.2 while 40.2% of the values were greater than 0.2 and 0.5% lacked sufficient information to determine precision (nulls) (Fig 2B). With respect to the ICC values for volume, we obtained point estimates of 0.93 (±0.02) and 0.9 (±0.01) where measurements were made by 2 and 3 operators respectively. These values decrease to 0.64 (±0.16) when 4 operators are considered (Fig 2C).

#### Accuracy: Volume vs weight comparison

**Fig 3:**
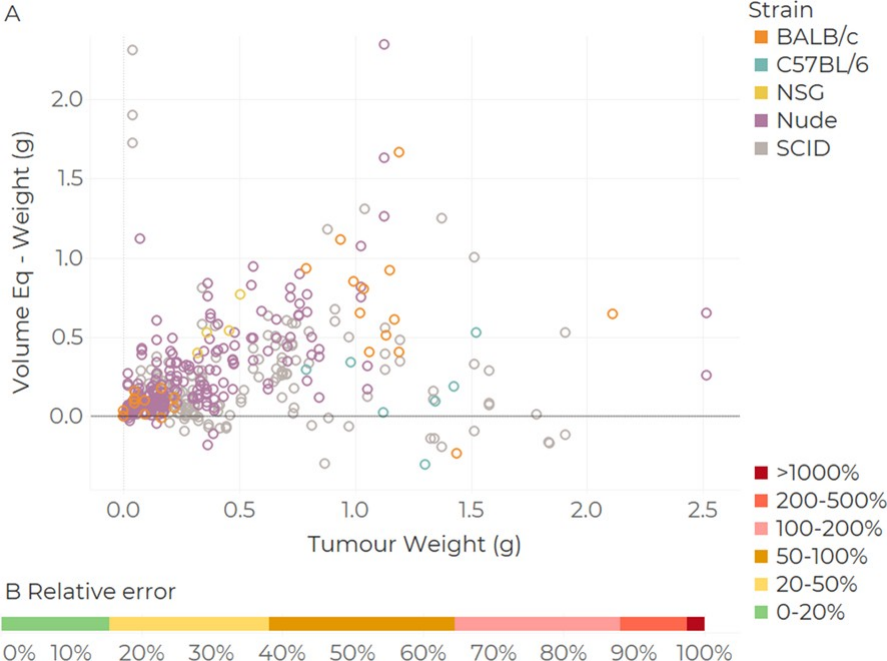
Tumour volume and weight comparison. Bland-Altmann plot (A), linear fit with 95% confidence intervals. Proportion of mice at different levels of relative errors (B, n = 440). Relativeerror=(VolumeEq.–Weight)/Weight.

Assuming that tumour density is $\rho_{tum}=1g/cm^{3}$, let us define a tumour volume equivalent to *V*_*Eq*_ = *V·ρ*_*tum⋅*_ We find that calliper-derived volume estimates exceeded excised tumour weight in 93.7% of cases (Fig 3). The distributions of relative errors between weight and volume shows that 29.0% of tumours weigh at least half the reported volume. In contrast, only 39.8% of weights exhibited relative errors of less than 50% and only 17.4% of comparisons returned errors of less than 20%.

### Cellular automaton model

#### Tumour morphologies

**Fig 4:**
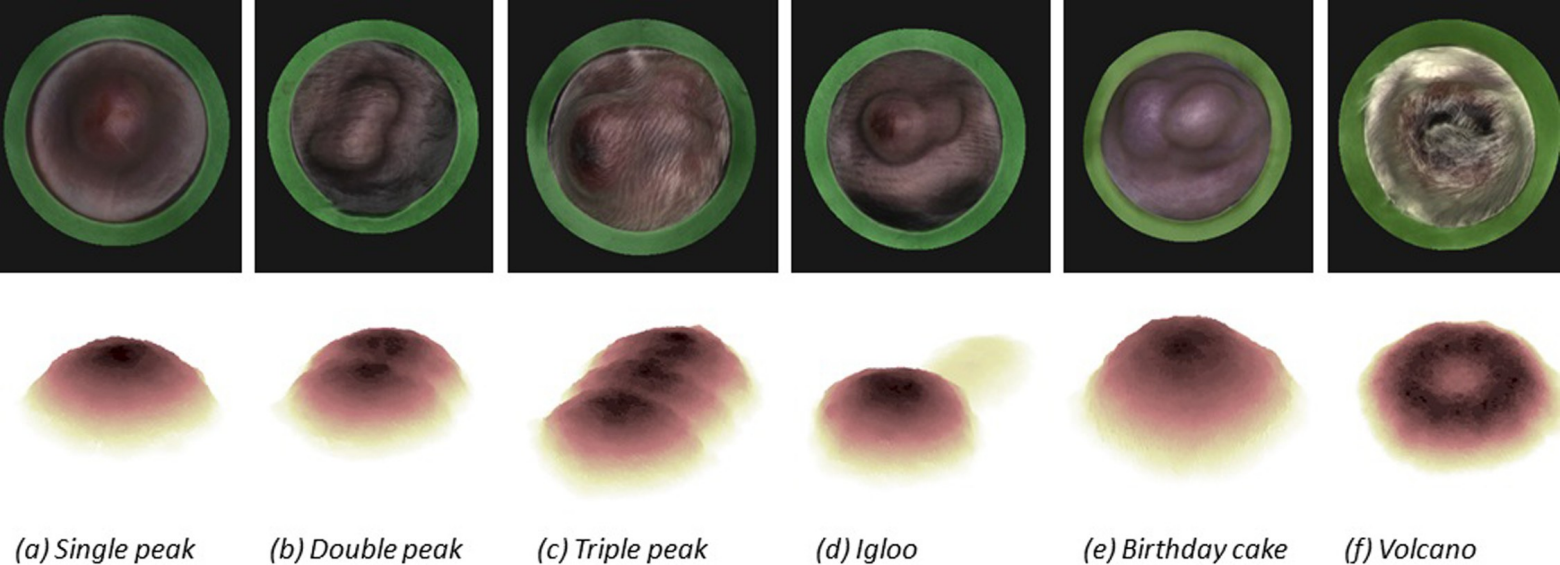
Representative examples of subcutaneous tumours exhibiting different morphologies. The top row shows reconstructions of real tumours produced using BioVolume. The bottom row shows snapshots of the corresponding in silico tumours generated with the CA model. a-c) depict tumours with one, two, and three peaks respectively. d) shows an igloo-shaped tumour. Such tumours are characterised by a main cancerous mass (typically resembling a single peak tumour) and a “tail” and can arise if the inoculating needle leaves a trail of cells when it is retracted. e) “birthday cake” tumours can be triggered by a mutation which creates a more aggressive sub-population of cells. f) volcano-shaped tumours can arise due to ulceration.

To assess the impact of using calliper measurements to estimate tumour volume we developed a Cellular Automaton (CA) model of tumour growth and its treatment (see Fig 4).

**Fig 5:**
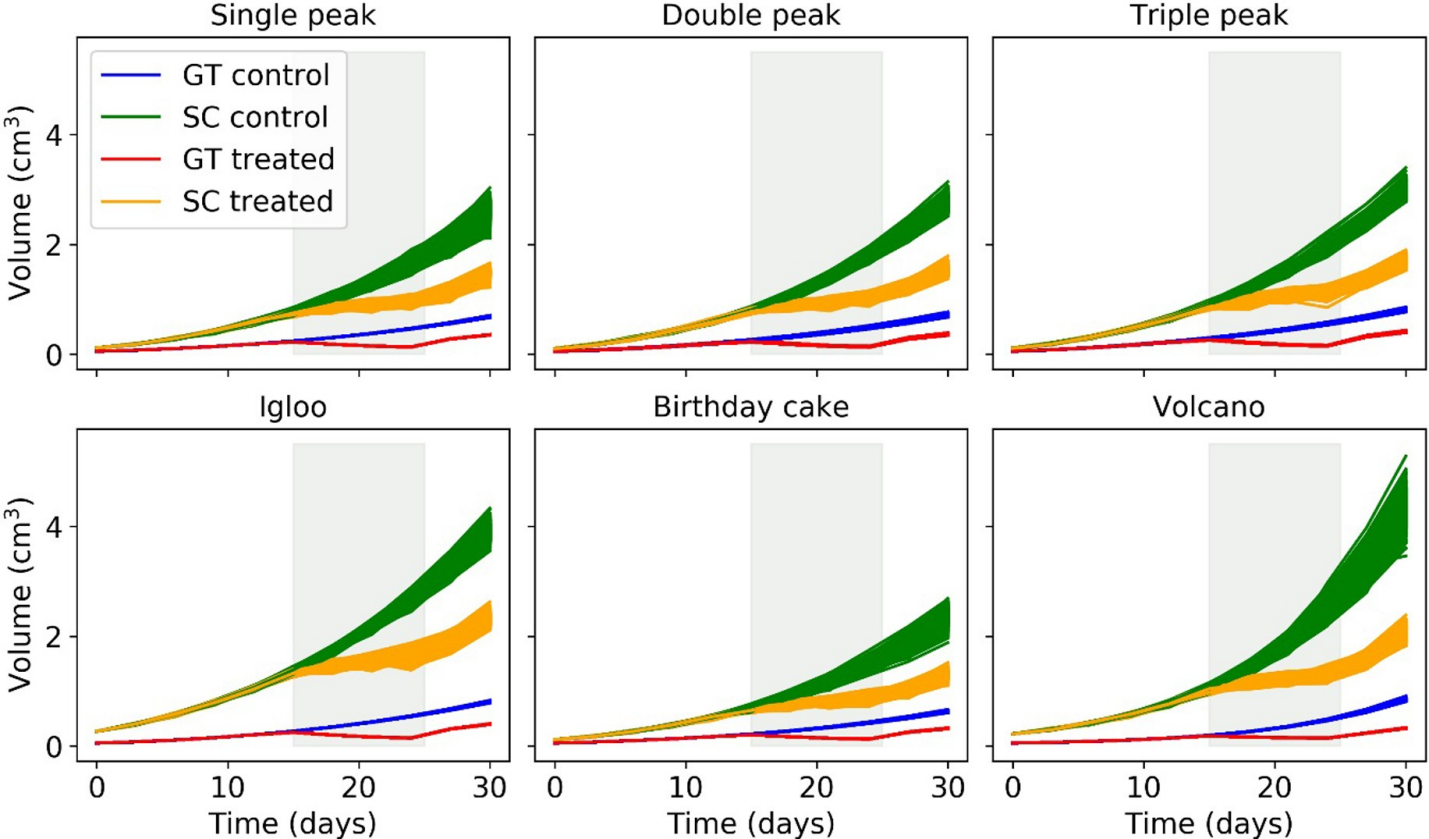
Growth curves of synthetic control and treated tumours calculated using the true volume (GT) and simulated calliper measurements (SC). For each morphology, 10^3^ growth curves were generated. The grey area corresponds to the period in which the anticancer treatment was applied; I.e. days 15 to 25.

We simulated the growth of both control and treated tumours for each morphology described in Fig 4. Treatment commenced on day 15 and lasted for 10 days. The tumours were subject to simulated calliper (SC) measurements which were then used to estimate the spheroidal volume of the tumour. These volumes were then contrasted to the actual tumour volume (computed as the sum of voxels), which we refer to as the Ground Truth (GT). The volumes of both control and treated tumours estimated from SC measurements were significantly larger and more variable than their GT volumes across all six morphologies (Fig 5). Visual inspection of growth curves for control and treated tumours indicates that the GT volume decreased during the treatment period for all six morphologies which was not captured by the SC growth curves.

#### Treatment efficacy metrics

**Fig 6:**
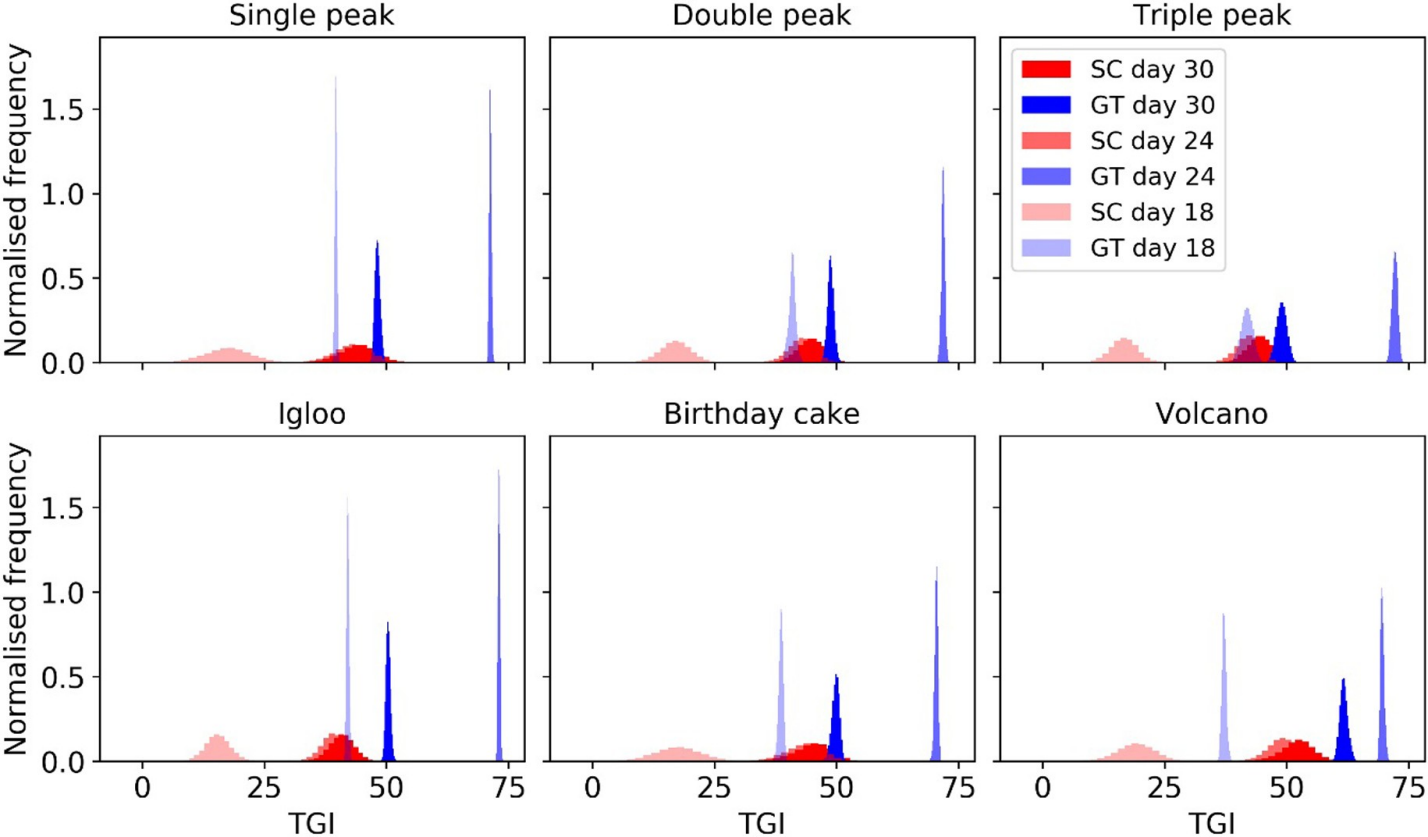
Histograms showing the Tumour Growth Inhibition (TGI) index computed for different morphologies using the true volume (shades of blue) and simulated calliper measurements (shades of red). The TGI was computed using days 18, 24 and 30 as experiment endpoints.

To determine the impact of simulated calliper measurements on the accuracy of treatment efficacy we calculated and compared two commonly used treatment efficacy metrics using the GT and SC-derived volumes. Specifically, we computed the Tumour Growth Inhibition (TGI) and Area Under the Curve (AUC) indices (see S2 Appendix for details). Fig 6 shows the histograms of the TGI indices computed for every pair of control and treated growth curves for each morphology on days 18, 24 and 30 (see S2 Appendix for histograms of combined morphologies). It is evident that the distributions for SC have a larger standard deviation and lower level of the effect, having effectively lower statistical power. The distributions are very significantly different, overlapping only minimally. The difference is most obvious in the early stages of the treatment (see Fig 6).

### Evaluation of BioVolume

#### Consistency between linear measurements

**Fig 7:**
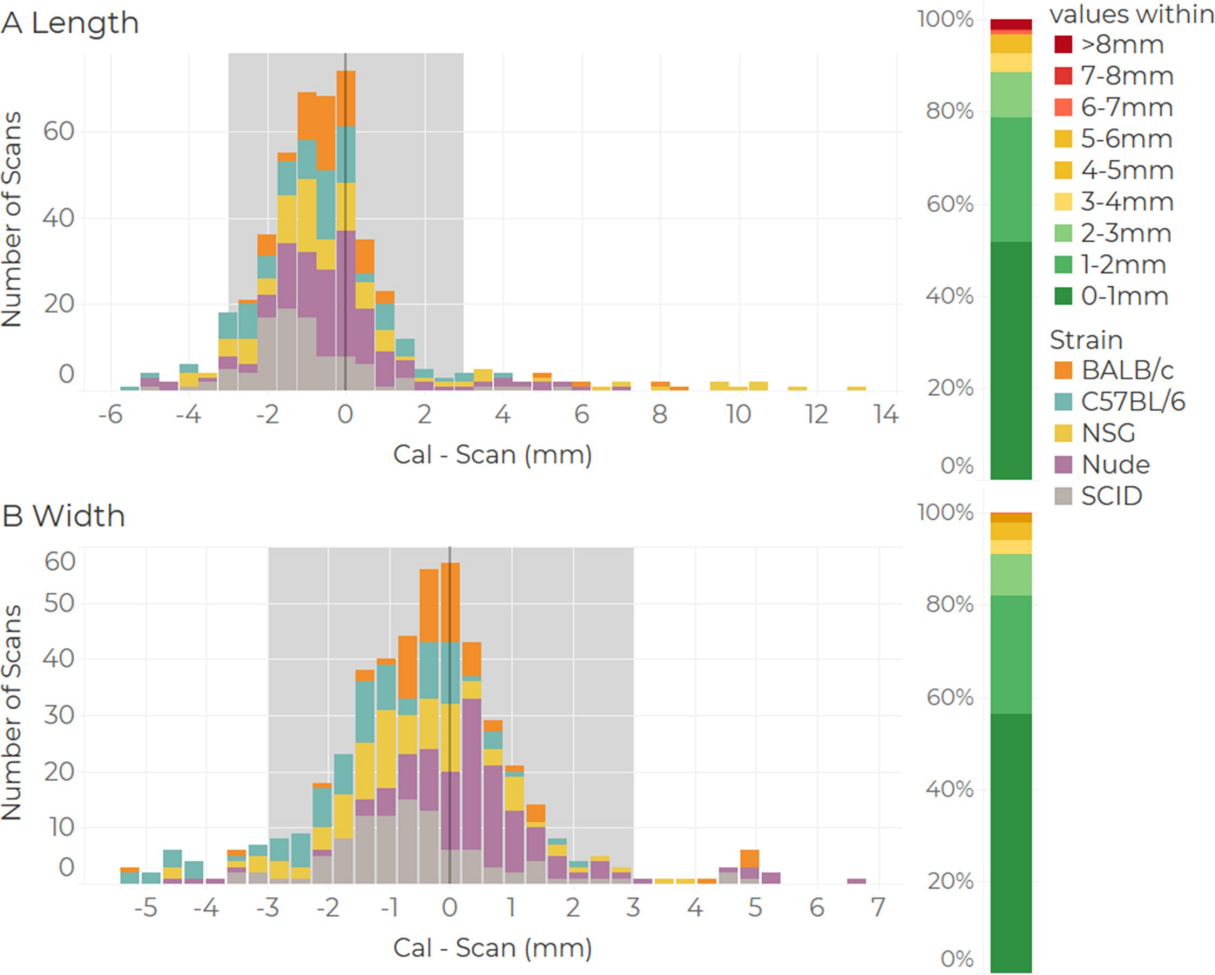
**Histograms showing counts of discrepancies between calliper and scan measurements (in mm)** for (a) length and (b) width of flank tumours. Counts within each bin are categorised by rodent strain. The vertical grey band highlights instance for which the difference between the scan and calliper measurement was less than or equal to 3mm. The vertical coloured bands to the right of each plot shows the number of scans falling into each range band as a percentage of the total.

We quantified the consistency between the linear length and width measurements made using BioVolume and callipers by making contemporary measurements of a given tumour using both methods and then, for each tumour, counting the number of scan measurements which fell within +/- 3mm of the calliper measurements made on the same day. These counts are displayed as histograms for both length (Fig 7A) and width (Fig 7B). Counts are organised into bins based upon the magnitude of the difference between the calliper and the scan measurements. The vertical grey band highlights values for which this difference was less than or equal to +/-3mm. The 3mm limit has been chosen as a representative figure of the standard deviation of the distributions (see S1 Appendix). We found the linear scan measurements to be highly consistent with those made using callipers. For length, 88.68% of the scan measurements fell within +/-3mm of their calliper counterpart and the same was true for 90.99% of width measurements. Furthermore, when we ranked mice by the colour of their fur, including white, black, and nude mice; where differences in performance are not significant (see Figure F in S1 Appendix for more details). Discrepancies greater than 8mm were only observed for 2.10% of length measurements and in 0% of cases for width. In these cases, the operator would have to outline the tumour region manually (which is a function incorporated in the system). Operators can identify errors in the segmentation by visualising the image on the scan data manager screen. When applying a t-test on distributions of length and width for both techniques, results are not significant with p-values of 0.330 and 0.148 respectively. Moreover, the symmetry and centrality at 0 of the Gauss bell shape of the distributions indicates a minimal bias in the linear measurements.

#### Volume—weight comparison

**Fig 8:**
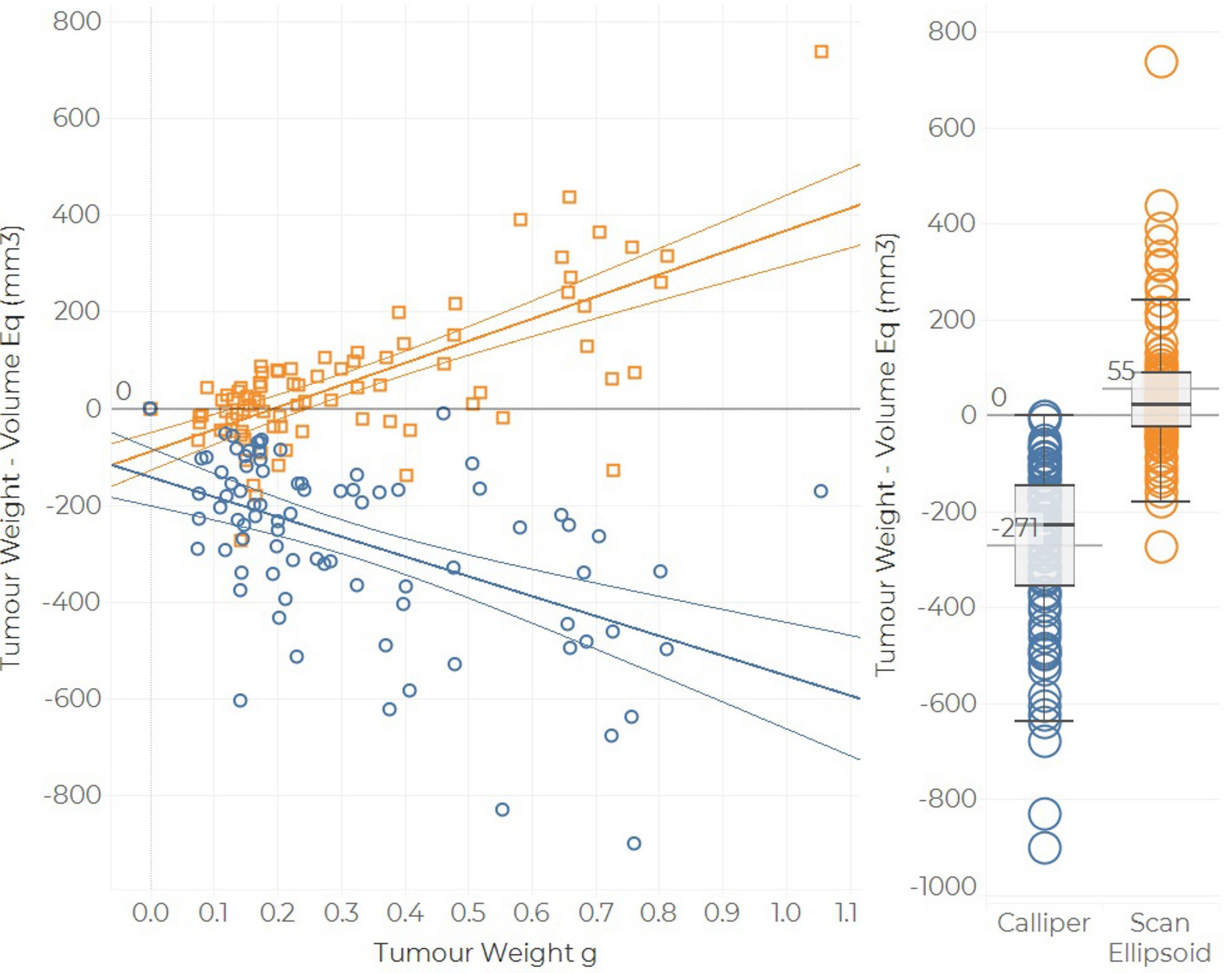
Volume—Weight comparison for Dataset 2. Calliper volume was calculated using the spheroid formula, whereas scan volume corresponds to the ellipsoid volume. The linear fit is represented by the solid coloured lines, whereas the horizontal gray line is the 0 reference line. The boxplots display the median discrepancies. The hinges of each box show the 95% confidence intervals and the whiskers extend to 1.5 times the IQR.

There are large discrepancies in the volume–weight correlations. Firstly, the scan ellipsoid formula (using length, width and height) shows both the smallest mean discrepancy (mean: 55 mm^3^, median: 24 mm^3^), the smallest bias (m = 0.499) and the best correlation coefficient (R ^2^ = 0.50, see Fig 8 and S3 Dataset), when compared to Callipers (mean: -271mm^3^, median:-227mm^3^, m = 0.411, R^2^ = 0.26). Notably, BioVolume displayed a systematic tendency to estimate lower tumour volumes than callipers, which corresponds to the lower values of observed weights relative to calliper-derived volumes observed in Fig 3 for Dataset 1.

#### Inter-operator variability

**Fig 9:**
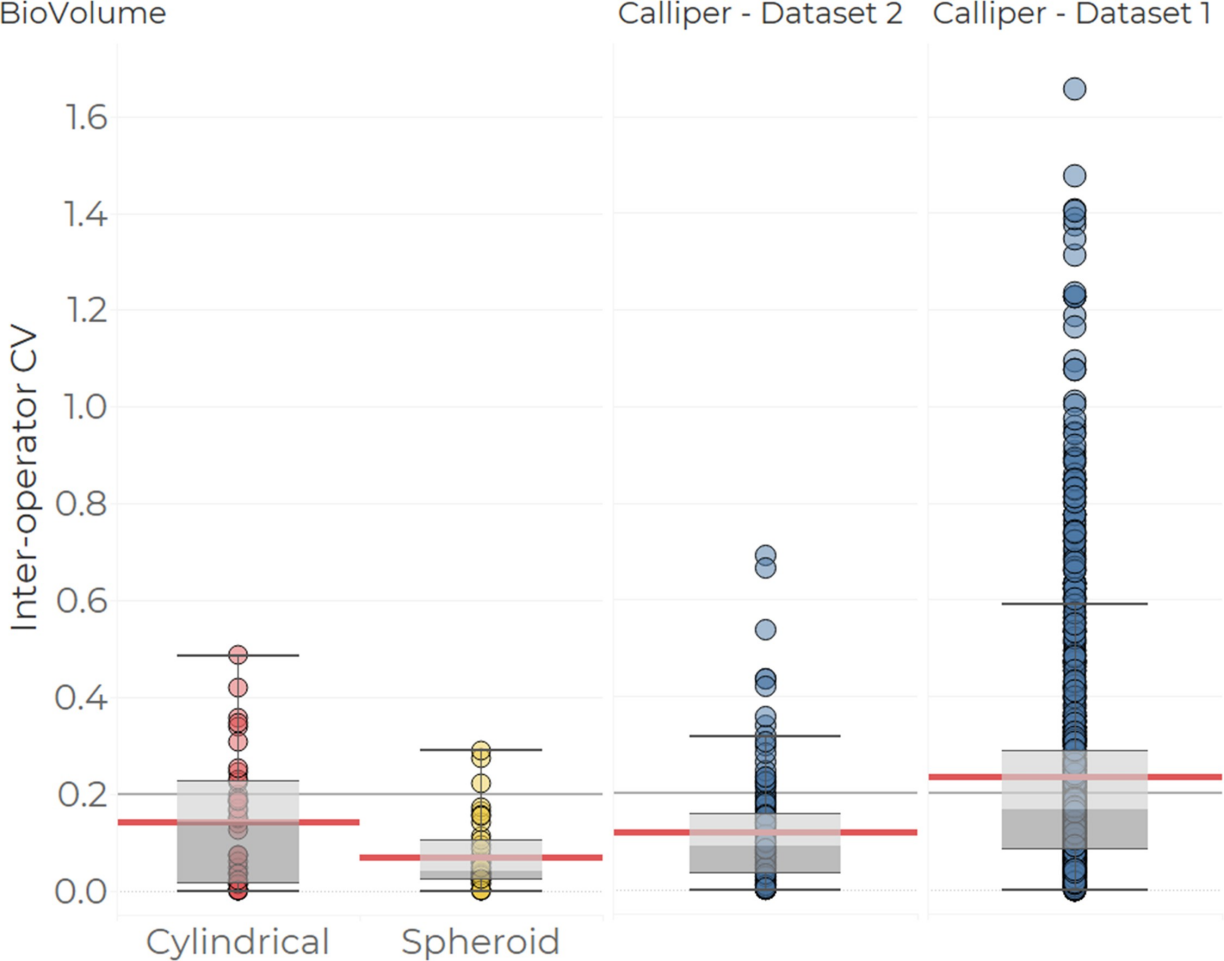
Inter-operator precision in volume estimates for callipers and BioVolume. Volume estimates for BioVolume correspond to spheroid (the same formula as that used for callipers, in yellow) and cylindrical approximations (in red). Calliper data (in blue) is split into values from the BioVolume evaluation (left) and values from the Calliper statistical review (right, also in Fig 2). Each point captures the inter-operator CV based on two or more volume measurements made for a specific tumour on a given day. The box plots summarise the dispersal of the estimates. The main body of each box highlights the inter-quartile range while the whiskers of each boxplot encompass all values within 1.5 of the median which is indicated by the dividing line between the upper and lower hinges of each box. The light red lines reflect the mean for each category.

We computed the inter-operator CV for the cylindrical and spheroid volume estimates derived from BioVolume’s measurements as well as for the calliper volume estimates from Dataset 1 (calliper statistical review) and Dataset 2 (BioVolume evaluation), see Fig 9. The inter-operator variability (CV) is a measure of precision, where CVs close to 0 indicate that repeated measurements of the same tumour produce similar values. Conversely, large CVs correspond to less repeatable measurements. Each point on Fig 9 reflects the variability between different repeats of the calliper or scan measurement on a specified mouse on a given day. We find that BioVolume’s spheroid estimates are more precise than those of callipers, whereas the cylindrical volume, which incorporates the height of the tumour, is comparable. There are large differences in precision between the calliper derived estimates from Dataset 1 and Dataset 2, with the former exhibiting greater spread and variability. 109 scans that were either misaligned or showed an error have been excluded (38% of Dataset 2). While the number of scans excluded is large, this dataset corresponds to an initial pre-production study during which the current version of the scanner was assessed for the first time. The scanner has undergone some significant improvements in the time since the evaluation, bringing the rates of misalignment and error down to approximately 15% as of June 2019 (results not shown).

## Discussion

In the calliper statistical review, we demonstrated that callipers are subject to high inter-operator variability, with values reaching 130% in inter-operator CV. Additionally, correlation between calliper-estimated volumes and excised tumour weight was poor (Fig 3). These metrics may compromise studies as tumour volume is used as a surrogate of tumour burden (weight). Furthermore, in Fig 3 we observed that there are larger discrepancies between operators for tumour models 4T-1 and A20. This may be partly because these models are known to invade tissues locally; and partly because they were implanted under the mammary fat pad; thereby exhibiting morphologies which are difficult to capture with callipers [18,19].

We selected a cellular automaton model to evaluate morphology since rules are easy to formulate and interpret as well as it being amenable to produce multiple morphologies with a degree of stochasticity. This is particularly interesting since we are aiming to characterise the span of multiple tumour shapes. Our computational model clearly demonstrates that assuming tumours to be spheroid -as when making measurements with callipers- is inadequate, particularly when tumours exhibit irregular morphologies. First, simulated calliper measurements failed to capture the decrease in tumour volume in response to treatment (Fig 5). Second, simulated calliper measurements reduced statistical significance when comparing treatment groups as demonstrated by the TGI and AUC indices (Fig 6). Thus, when using callipers, there is an increase in the variance of measurements and a significant shift in the group mean which is sensitive to morphology (see S2 Appendix). The most important model parameters were the vertical bias and the rate of cell division *p*_*divi*_ (see the sensitivity analysis in S2 Appendix). Moving forward, the model could be extended to account for different local invasion scenarios and more complex pathophysiological factors by modifying these parameters to be time-dependent. This would make the model more reflective of more complex tumour models such as syngeneic and Patient-Derived Xenografts (PDXs).

Using the prototype BioVolume scanner we were able to replicate calliper length and width measurements to within +/- 3mm in around 90% of cases (see Fig 7). Thus, both techniques produce comparable linear measurements. Volumes estimated using BioVolume were highly correlated to those estimated with callipers but were lower on average. As shown in Fig 8, BioVolume’s estimates correspond more closely to excised tumour weight than callipers. This implies that BioVolume will provide a better estimate of weight than callipers, primarily because BioVolume measures height whilst callipers incorrectly assume height to be equal to width. This effect was explored in the modelling section of the paper. The current approach to volume—weight comparison can be challenged as it cannot be used in longitudinal studies. Additionally, it rests on significant assumptions such as: a constant/translatable value of density, homogeneous tumour consistency, and consistent operation (some tumours may present with significant amount of fluid that may leak out upon excision). Despite these results being positive, further work needs to be performed to validate the performance of BioVolume. Specifically, direct comparisons with imaging techniques such as US, MRI or CT would provide useful insight into the accuracy of BioVolume’s estimates.

Finally, the inter-operator variability of BioVolume outperforms that of callipers when using the spheroid formula for volume estimation. When tumour height is introduced (via the cylindrical formula, see Fig 9 and Figure J in S1 Appendix) inter-operator variability is comparable between callipers and BioVolume. This is in part due to the fitting of the back of the mouse, the fitting of the plane to find the normal, and the choice of the top point. Small variations in any of these aspects will negatively impact the consistency of height measurements. In future versions of BioVolume, we aim to improve upon these issues and to introduce a volume calculation based upon the integration of the complete surface of the tumour. We excluded 109 (38%) anomalous scans from our analysis. These anomalies arose due to system errors, misalignment of the thermal/RGB images, or motion blurring. Work is ongoing to prevent such occurrences in the future by i) improving the robustness of the code and ii) by improving the training protocol provided to experimenters using BioVolume.

BioVolume, in its current form, presents a promising alternative to callipers. It has the scope to provide accurate measurements with reduced human bias. Furthermore, measurements are traceable and calibrated, as images can be revisited at any point post-capture and measurements extracted manually if required. Images can also be inspected by other users who are logged onto the system remotely potentially easing communication, peer-review, and cross-validation. Additional work is underway to improve BioVolume’s performance and to expand its functionality. For example, machine learning can be applied to classify and characterise the stored tumour images, potentially offering additional biomarkers for treatment efficacy/toxicity and for animal welfare.

## Conclusion

In conclusion, the use of linear calliper measurements for tumour volume estimation in lab animals is subject to significant accuracy and reproducibility problems which negatively affect the power of preclinical studies and animal welfare. We proposed BioVolume as an alternative to callipers which provides non-invasive, traceable, and more reproducible measurements with the potential to be fully morphology-independent and to surpass callipers’ performance.

## Supporting information

S1 Appendix Supplementary materials for calliper statistics & BioVolume. (DOCX)

S2 Appendix Supplementary materials of Tumour Growth modelling. (DOCX)

S1 Dataset Structured data for calliper analysis. (CSV)

S2 Dataset Structured data for calliper and BioVolume comparison. (CSV)

S3 Dataset Structured data for the Calliper and BioVolume weight comparison. (CSV)

S1 Scan An example of a scan. (7Z)
