# Supplementary material for: An innovative non-invasive technique for subcutaneous tumour measurements
Source: PLoS One. 2019 Oct 14;14(10):e0216690. doi: 10.1371/journal.pone.0216690 (PMC6791540; doi:10.1371/journal.pone.0216690)
Supplement: S1 Appendix — (DOCX) [file pone.0216690.s001.docx]

**S1 Appendix - Supplementary Materials for Calliper Statistics & BioVolume**

**From paper:**

Delgado-SanMartin et al. An innovative non-invasive technique for subcutaneous tumour measurement, submitted to PLOS ONE, 2019

# Additional Background

Animal models of human cancers are fundamental to our understanding of tumour biology. Pre-clinical studies remain essential to extend our understanding of the mechanisms responsible for cancer and to identify, for example, new targets for biomarker discovery and validation. In drug discovery, the use of such tumour models is important in screening and testing potential new anti-cancer therapies and they continue to be useful in translational studies which support new cancer drugs in clinical trials.

Many different tumour models are employed in pre-clinical oncology research where they act as surrogate models for the human disease. Tumour models for pre-clinical research may be derived from either human or rodent tumour cell lines and, more recently, from patient derived tumour tissue. Tumour models are often classified by implant site, such as sub-cutaneous xenografts which are often implanted onto the flank of mice and orthotopic xenograft models where tumours are grown in the tissue of origin. Tumour volume is a significant metric in preclinical trials where it provides a surrogate for both disease progression and treatment efficacy. Thus, accurate and repeatable estimation of tumour volume is crucial if we are to be able to declare a given trial to be a success or failure with confidence [1]. At present, it is standard practice to estimate murine tumour volume by manual measurement of tumour length and width using callipers. However, the continuing persistence of callipers is in spite of their long-established susceptibility to inter and intra-operator variation [2]. The precision (a measure of repeatability) and accuracy (the extent to which the measurements reflect the true dimensions of the tumour) of calliper measurements is fundamentally undermined by the complexity of the tumour surface: tumours are irregular, compressible and mobile beneath the skin [2]. Thus, the act of manually measuring a subcutaneous tumour deforms it and this deformation is sensitive to the idiosyncrasies of individual operators [2]. That is, variation in handling and measurement styles may translate into variation in measurements. An additional complication is the use of ellipsoidal formulae to estimate tumour volume. These formulae provide the most robust estimates of tumour size [1] but they require measurements of three distinct axes (length, width and height) of which only two, length and width, are tractable to callipers. As such, a spheroidal approximation is frequently used in which width and height are commonly assumed to be equivalent, introducing an additional confounding factor when this assumption is violated.

The systemic inaccuracies associated with callipers can lead to high levels of intra and inter-operator variability [2]–[4]. This has several significant consequences for how pre-clinical trials are conducted and interpreted. First, it inflates the number of mice which must be committed to trials to provide sufficient statistical power to detect treatment effects [2]. This has implications for both animal welfare and the cost efficiency of pre-clinical trials. Second, the most common approach to minimising the effects of high inter-operator variation is to simply assign only a single operator to each study thus leaving only intra-operator variability to be accounted for [2]. However, this is a crude solution which significantly constrains the design and scheduling of trials. In particular, that measurements made by distinct operators cannot be treated interchangeably may lead to non-trivial complications in the event of, for example, operator absence.

In recent decades, non-invasive technologies for the accurate determination of subcutaneous tumour volume have become increasingly available [3], [5]–[7]. Comparison studies have demonstrated that Ultrasound (US), MRI and CT scanning all produce volume estimates which are both more reproducible than those of callipers and which are more sensitive to treatment induced changes in tumour size [3], [5]–[7]. However, the use of these technologies is fundamentally constrained by their impracticality; they require extremely specialised, expensive equipment and the animals must often (but not always, see Kersemans et al. 2013) be anaesthetised or otherwise restrained which calls animal welfare into consideration when repeated measurements are to be made [4], [8], [9]. That callipers have yet to be replaced, in-spite of both the availability of accurate non-invasive methods and their known deficiencies, is a testament to how highly their affordability and ease of use are valued.

Thus, there is a need for a non-invasive means of assessing subcutaneous tumour volume that is affordable, portable and which allows for the animal to be awake and only lightly restrained. In this regard, the potential of 3D and thermographic imaging remains underutilised. While less accurate than US or MRI, these technologies still offer the scope for significant gains in precision and sensitivity relative to callipers whilst also being more affordable and portable than US or MRI [4], [8], [9]. Modern 3D scanning methods provide the opportunity to non-invasively derive key tumour metrics such as length, width and -crucially- height [4]. This valuable spatial information affords experimentalists the capacity to represent tumours as complex, irregular surfaces that we know them to be, expanding the means by which tumour size and volume may be estimated beyond the classical spheroidal and ellipsoidal approximations. Thermography is well established as a means of detecting breast cancer in humans, where inflammation renders the tumour warmer than the surrounding tissue [8], [9]. A similar, yet opposite, thermal contrast is observed in mice bearing human-derived xenografts: xenograft tumours tend to be cooler than the surrounding tissue [8]. Consequently, it has previously been established that thermography can utilise this temperature differential to detect subcutaneous tumours in lab mice [8]. Furthermore, thermal imaging is sufficiently sensitive to detect developing tumours before the manifestation of physical signs and can also detect responses to some therapies within hours of exposure [8], [9]. By utilising the thermal contrast between the tumour and its local environment, thermal imaging simplifies the accurate delineation of tumour boundaries, reducing ambiguity regarding the extent of the tumour.

The benefits offered by 3D and thermal imaging are distinct, yet potentially complementary. The benchtop scanning solution BioVolume integrates both 3D scanning and thermal imaging technologies to generate accurate 3D reconstructions of subcutaneous xenograft tumours and to estimate both tumour area and volume. BioVolume enjoys the key benefits of both technologies: measurements can be made on awake, lightly restrained animals, without mechanically disrupting the tumour. Thus, BioVolume may represent an affordable alternative to MRI, US and CT which can treat tumours as spatially complex objects whilst also circumventing the inter-operator variability associated with callipers.

# Methods

## Experiments

### Animal models

A variety of animal models have been used in the making of this paper, although no studies have been specifically designed to test the BioVolume unit. As mentioned in the main text we explored two datasets:

- Dataset 1: Calliper statistical review

Records for 1,608 mice and 2,488 calliper measurements were collected by AstraZeneca over a period of 17 months from February 2017 until June 2018. Calliper measurements were acquired by 29 operators. We used 31 tumour models grouped by organ of origin, Lung, Breast and Colon being the most popular (see Figure A).

- Dataset 2: Scan evaluation

Additionally, we present an evaluation dataset collected on 3 occasions between 14/09/18 and 05/10/18 using both callipers and BioVolume. A total of 257 calliper measurements and 297 scans were taken (see Figure B). Images were acquired by 4 operators. Six known tumour models were used while for 61 mice, information regarding the tumour cell line was not available.

Experiments have been carried out at two distinct AstraZeneca locations (Alderley Park and Cambridge). Both establishments comply with UK Home Office licences for laboratory animals. Animals were caged in 114ST standard cages, grouped into fives and then racked in TN72 model rails. Husbandry practices were compliant with AWERB and ethical / scientific review. Calliper measurements were taken using calibrated digital manual callipers.

The data corresponded to 43 in-house studies for different cancer drugs and growth curve characterisation. The extent of these treatments and experimental designs is beyond the scope of this paper, therefore full disclosure of treatment details will not be provided. All cell lines were sourced by ATCC (American Type Culture Collection) some of which were modified. The patient-derived xenografts were sourced from patients in the US (AstraZeneca’s GHP site). We used 5 mice strains (SCID, BALB/c, C57/BL6, NSG and Nude) sourced by Charles River UK ([www.criver.com](http://www.criver.com)) & Envigo UK ([www.envigo.com](http://www.envigo.com)).

Figure A: summary of the calliper data (dataset 1). Proportions of data divided into tumour model, strain, gender, size, system errors and numbers across time. Colours of the bottom panels correspond to animal strain.

In the Dataset 2, we were able to image tumours that were too small to be palpated or measured with callipers, referred to here as “pre-palpable” (15% of the dataset). We experienced system failures in 9% of cases, the causes of which are explained in Figure B. For Figure 9 only on the main paper, we excluded scans with another form of error: misalignment of RGB/thermal images or when images have moved/blurred. This occurred in 40% of the cases. Finally, we also investigated orthotopic tumours which were implanted into the mammary fat pads. These tumours constituted 12.5% of the collected images.


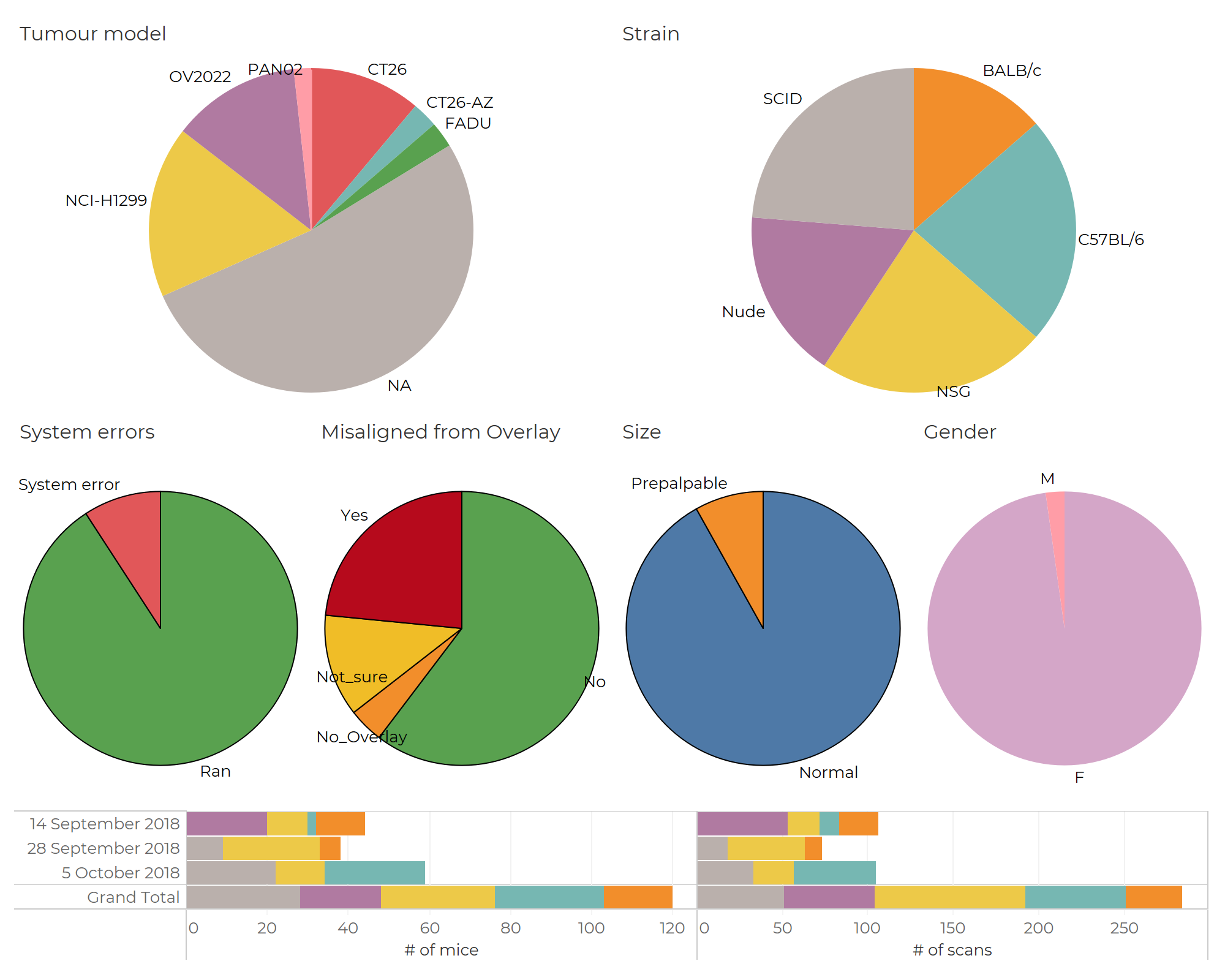


Figure B: summary of the BioVolume evaluation data (dataset 2). Proportions of data divided by tumour model, mouse strain, mouse sex, tumour size, system errors and numbers of mice and scans across the three occasions (days 5, 14 and 28). Colours of the bottom panels correspond to animal strain.

### Data collection protocol

Upon acquisition of the data the technician would collect the required cage and place it on the benchtop. Mice would be handled one by one, using their earmarks for identification. Once identified, the weight of the mouse would be recorded using a standard balance. Subsequent to weighting, the mouse would be held again and restrained such that the tumour region was exposed to the operator. The operator would then measure the maximum length orthogonal to themselves and take an approximately 90-degree angle measurement of the width. In cases where tumours were irregular or deformable (e.g. due to oedema), the maximum width would be recorded. For image acquisition using BioVolume, the mouse would be held against the aperture of the device, and the acquisition button triggered. Hairy mice were shaven in advance. Proper shaving is fundamental for the success of image acquisition as hair insulates the skin and scatters infrared light, concealing the temperature difference between the tumour and the surrounding tissue.

### BioVolume image acquisition

The BioVolume hardware acquires both photographic (a.k.a. red-green-blue, RGB images) and thermal (infrared) images. The RGB images are fed into a reconstruction engine to create depth and colour maps. The thermal images are used for segmentation: the outlining of the tumour region or mask. The resulting mask is then co-registered onto the depth and colour maps. Morphometric values such as length, width, height, area and volume are obtained from this mask (Figure C). While we do not disclose the full details of the underlying algorithms, we provide a top-level outline of the relevant processes (Figure C).


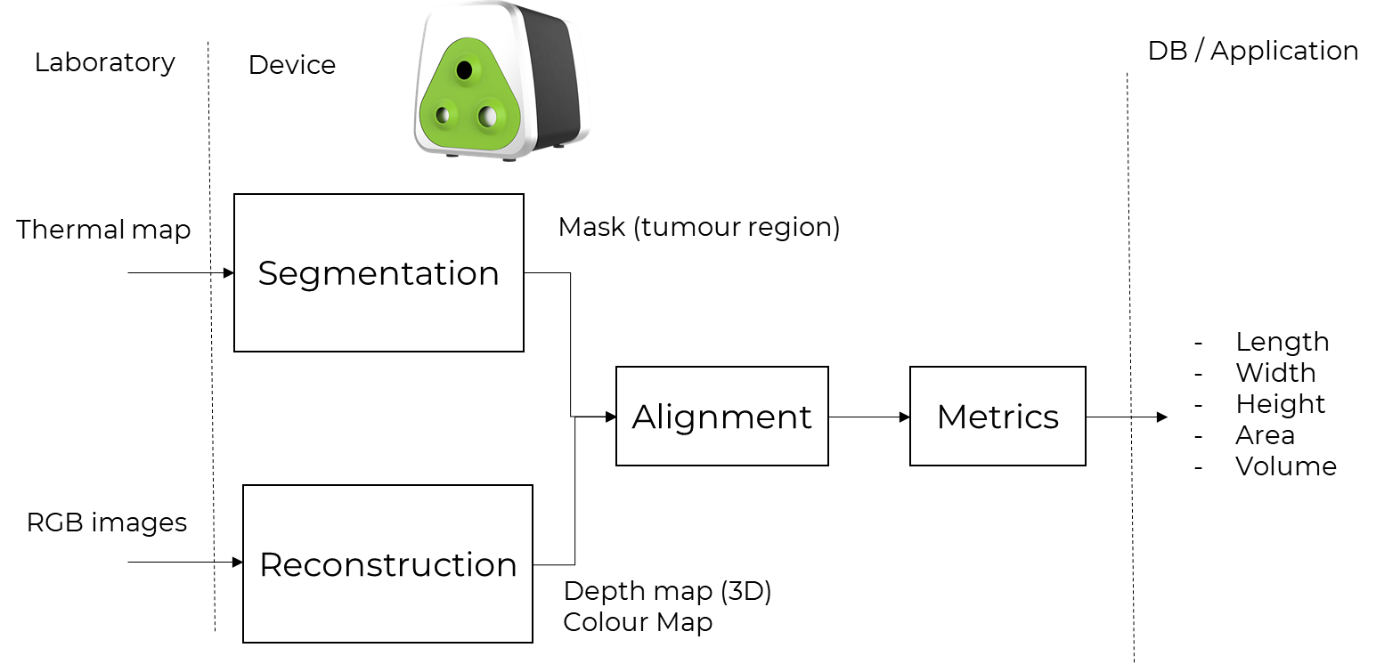


Figure C: Software and image processing pipeline.

Our proprietary 3D stereo reconstruction engine is a unique application developed in-house at Fuel3D. Depth is calculated using algorithms based on piece-wise variational correspondence of the RGB images [10], [11]. The depth is then to inform the geometry of the system (the positioning of the cameras and the distance between the aperture and the optical systems) and for the acquisition of intra-image information.

At present, segmentation is performed by applying a polar coordinate search path algorithm, which is related to active contour segmentation, to the thermal map [12]–[14]. In preparation, we exclude cold areas by applying a threshold at 70% of the of the range between min and max temperature values i.e. below $T_{min}+0.7\cdot\left( T_{max}-T_{min} \right)$ is removed outside of the mouse. The segmentation requires a seed point to be specified within the tumour area, around which the image is transformed into polar coordinates., The boundary of the tumour is then given by a function $r\left( \theta\right)$, with the constraint $r\left( 0 \right)=r\left( 2\pi\right)$. In the discretised image, the contour is represented by radii at fixed angular intervals: $r_{1}, r_{2}, \ldots r_{n}$. In this case the energy functional is defined to be the average gradient (of the image) on the contour, $\sum_{\left\{ i=1 \right\}}^{\{n\}} \nabla I\left( \theta_{i},r_{i} \right)$, which is then maximised subject to the constraint $\left| r_{i+1}-r_{i} \right|\leq\epsilon$ to enforce continuity. This is implemented by finding the optimal path through a graph, with weights given by the gradient, and continuity enforced by the presence (or absence) of edges.

Once the tumour has been segmented, the mask is co-localised onto the 3D map. The projected region is then excluded and cold areas are removed further to include the back of the animal only. The threshold here is 50% of the range between min and max temperature value i.e. below $T_{min}+0.5\cdot\left( T_{max}-T_{min} \right)$. An iterative optimisation algorithm then fits the back of the mouse onto a parametric quadratic surface. To achieve this, we use a pseudo-linearisation of the quadratic surface and solve the linear system $A\cdot x=b$. Put simply, $x=A^{*}\cdot b$, where $A^{*}$ is the pseudo-inverse Hermitian matrix of coefficients calculated by singular value decomposition. The process is carried out iteratively using a RANSAC type approach whereby outlying points from the back of the mouse are excluded, and new points close to the modelled surface are included before fitting a new surface. The tumour mask is subsequently projected onto the quadratic plane and these points are fitted to a linear plane, the normal of which determines the direction of the height of the tumour. The length, width, area and height of the tumour are then calculated. Length and width are extracted from the 3D projection onto the quadratic plane by rotating the figure until its coordinate system aligns the longest dimension of the mask to the longest dimension of the bounding box. The width is then the shorter dimension of the resulting bounding box. The area is the projected area on the 3D linear plane. We propose three different calculations of the volume:

- Spheroid: $V_{sph}=\frac{\pi}{6}\cdot length\cdot width^{2}$ (to mimic callipers)
- Ellipsoid: $V_{ell}=\frac{\pi}{6}\cdot length\cdot width\cdot height$
- Cylindrical: $V_{cyl}=Area\cdot height$

### Code & Software

We used a variety of scripting methods in this work. Specifically, the following software was used:

- 3D reconstruction, alignment: C++ (on MS Azure)
- IR image segmentation: C++ (on MS Azure)
- Statistics & Data Analysis (calliper data): R (packages: psych, latticeExtra, ggplot2, dplyr, reshape2) & Python (packages: stats)=)
- Cellular automata model: Matlab (packages: statistics)
- Figures (calliper and BioVolume data): tableau 3.1

The plots were made querying an internal Fuel3D MySQL relational database hosted by Amazon Web Services (AWS). The code for the cellular automaton is provided also in the supplementary materials. The rest of the code is proprietary and withheld at Fuel3D’s discretion.

## Statistical analysis

In this paper we used a number of statistical analyses. For the calliper statistical review (Dataset 1) see sections 2.2.1 and 2.2.2 and for the BioVolume evaluation statistical review (Dataset 2) see sections 2.2.3 and 2.2.4.

### Precision: Inter-operator reproducibility

We use two metrics to assess inter-operator repeatability: the coefficient of variation (CV) and the intra-class correlation (ICC). A lower CV and a higher ICC are preferred.

Precision is a measure of dispersal that illustrates the repeatability of a given measurement method. For a chosen measurement method, the precision of the measurements made for a specified tumour on a given day is found as the standard deviation of all such measurements (across all operators) for that tumour on that day, divided by the mean of those measurements. We defined it as: $CV_{operator}=\frac{\sigma}{\mu}$, where $\sigma$ denotes standard deviation and $\mu$ denotes the mean between measurement of different operators. Thus, values close to 0 indicate consistent inter-operator measurements while greater values indicate inconsistencies between operators.

The Intra-class correlation (ICC) was calculated contrasting the between-mice variation and within-mice variation. In the reproducibility part of our experiments, each mouse was measured by *k* operators (where *k is equal to 2, 3, 4, 5 or 6*), which were selected for each mouse from a larger population of operators (in contrast to the same *k* operators measuring all mice).Consequently , we were able to fit a one-way ANOVA to decouple between-mouse variation and the within-mouse variation which were estimated by the between-mouse mean squared (BMS) and the within-mouse mean squared (WMS) respectively. The ICC is then defined as

$$ICC=\frac{BMS-WMS}{BMS+\left( k-1 \right)\mathrm{WMS}}.$$

An extensive discussion of ICCs and a more detailed definition can be found in [15].

### Volume – weight comparison

We use excised tumour weight as an extrinsic ground truth value. However, only a limited number of weight values (439) were collected. Weights were collected by weighing tumours using a precision balance post-excision. During excision, attempts were made to maintain tumour integrity whilst discarding extra skin and peritumour fibrotic tissue. Oedema and fluid bags were not collected due to fluid being difficult to collect.

It is common practice to compare the weight of the excised tumour to the estimated volume, assuming constant density. The density values of human soft tissue ranges from 0.9 (fat) to 1.09g/cm^3^ (skin) [16], and tumour densities have been studied extensively with a consensus density of 1g/cm^3^, i.e. that of water. Given these assumptions, we calculated the discrepancy between volumes estimated using calliper measurements and excised tumour weight with the formula $Volume Equivalent =Density\cdot Volume$, in grams.

### Calliper – Scan linear consistency

We use the consistency between the linear measurements of callipers and BioVolume as a soft surrogate metric for accuracy. No reliable metric of ground truth (such as an MRI scan) was available and therefore we cannot assess the true accuracy of either technique. Instead, we can only compare them against each other. We used a fixed acceptance threshold of +/- 3mm. This reference value is an approximation of 1.5 of the interquartile range (IQR) for the distributions of both length and width. The actual values for the IQR were 3.12mm and 2.64mm for length and width respectively. For length, the standard deviation was 2.96mm while for width it was 2.27mm.

### Statistical analysis: ANOVA

When designing efficacy studies in preclinical oncology, the most commonly utilised test is an analysis of variance (ANOVA). The exact details how to run the ANOVA (e.g. relative or absolute tumour volume, across time or at one timepoint, etc) is a frequently discussed topic which is beyond the scope of this work. Our ANOVA compared absolute tumour volume between the control and treated groups at the end of the study. We calculated the tumour volume for callipers using the spheroid formula (see Section 2.1.2).

We compared the mean log tumour volumes of the control and treated groups at the final time point. Statistical significance was evaluated using a two-sample, one-sided t test with unequal variance. We adjusted for multiple testing using Benjamini & Hochberg’s control of the false-discovery rate [17].

Although, we have a varied dataset with respect to inter-operator repeats, mouse strains and tumour sizes, we lack for information regarding treatments and control groups which prevents the plotting of growth curves. Therefore, we again use the CV as it can be calculated independently of how groups are defined. The $CV_{groups}$ is a function of the t- statistic used in the conventional ANOVA method for significance and power analysis.

# Supplementary Results

In this section, we outline additional results obtained when comparing callipers and BioVolume.

## Additional achievements of BioVolume

### Pre-palpable tumours

Very small tumours, too small to properly palpate with callipers are common during early stages of a study, shortly after inoculation, as well as when a treatment is effective and the animal enters remission. We refer to these tumours as pre-palpable. BioVolume can detect the thermal signature of these tumours (see Figure D). The automatic segmentation was successful for 8/16 of the observed pre-palpable tumours (above 50% of co-localisation of the predicted tumour region with the manually outlined tumour region). Our chosen metric for this was the DICE coefficient, which we defined as $Dice=\frac{2\cdot A\cap B}{A\cup B+A\cap B}$, where A and B are the predicted and manually outlined sets. The rest of images were able to be manually segmented. This implies that studies can be started earlier or, in the case of complete remission, continued for longer.


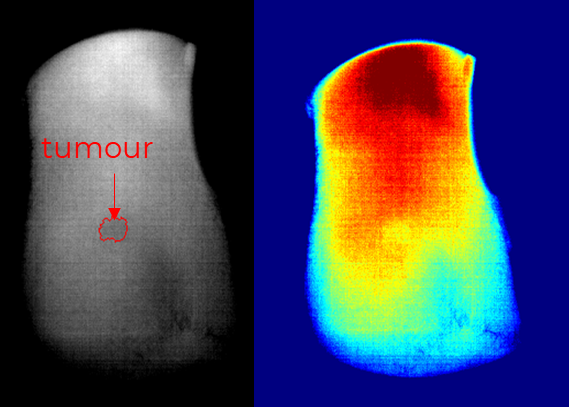


Figure D: Example thermal image of pre-palpable tumour. The tumour is under 3mm in diameter. We show the thermal map in grey and jet colour scales for comparison.

### Orthotopic tumours (Mammary fat pads)

As part of the BioVolume evaluation study, we included 85 independent measurements of orthotopic tumours, specifically breast cancer tumours implanted into the mammary fat pad. The results (Figure E) show that 82.2% and 93.1% of the measurements fall within +/- 3mm of calliper measurements for length and width respectively.

Figure E: Histograms for mammary fats pad tumours showing counts of discrepancies between calliper and scan measurements (in mm) for (a) length and (b) width of flank tumours. The vertical grey band highlights instance for which the difference between the scan and calliper measurement was less than or equal to 3mm. The vertical coloured bands to the right of each plot shows the number of scans falling into each range band as a percentage of the total.

### Performance on animal appearance

We have evaluated the performance of the algorithm for different animal appearances using length and width and determined that for both length and width each animal appearance performed very similarly with 85% to 93% of points lying within +-3mm of calliper for any mouse strain (see Figure F).


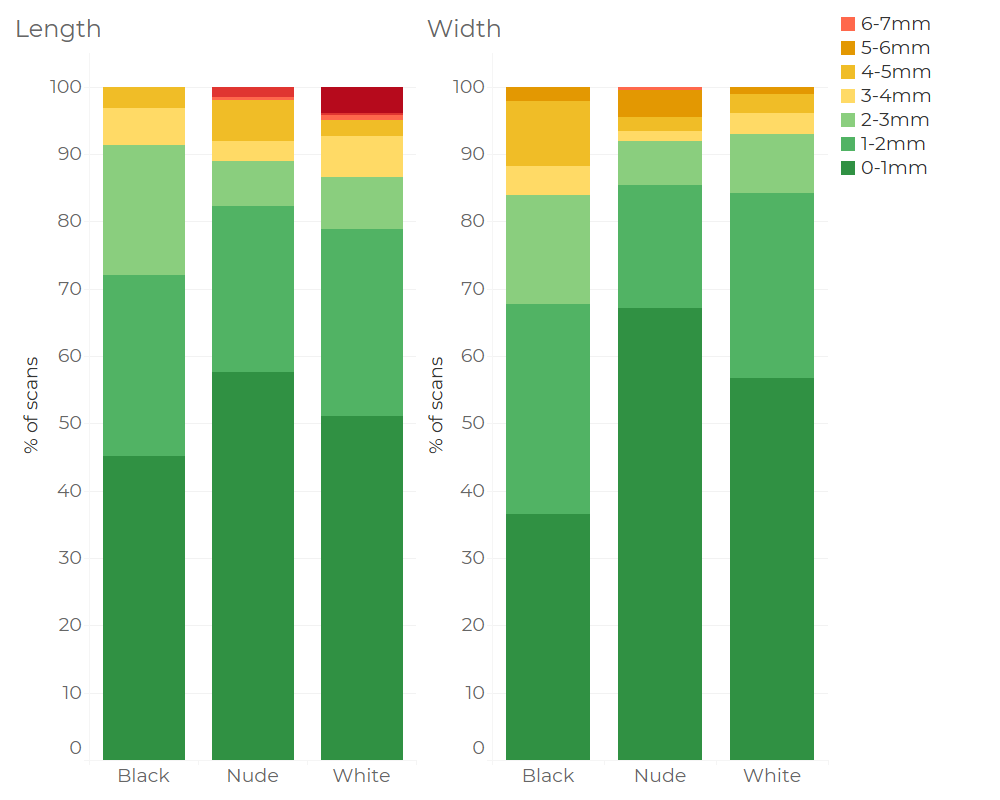


Figure F: agreement between scan and calliper length and with broken down by animal appearance.

### System & technical errors

This BioVolume prototype evaluation was used to examine some possibilities and different configurations of the hardware and software. For this reason, we excluded data points which fell under these criteria:

- tumours for which the seed fell outside the tumour region,
- unexpected system errors.

For Figure 9 of the main text only we excluded:

- tumour images that were impossible to align,
- mouse moved between flashes.

## BioVolume affects study outcome

For tumour volume estimates made using both callipers and BioVolume, we performed a simple ANOVA to compare the tumour volumes observed for the control and treated groups on the last day of dosing (see methods section). Figure G shows the results for an AZ study on 39 female Balb/C mice with CT26 tumours. The ANOVA shows that both callipers and BioVolume produce estimates which identify doses 2 and 3 as being significantly different from the other treatments nine days post-dosing (Table A).

Table A: Analysis of variance to compare tumour volume between the control and treated groups at the last day of dosing for callipers and scan (see methods section). Asterisks denote statistical significance, more asterisks more significance.

| Method | compare | p-value | Sig |
| --- | --- | --- | --- |
| calliper | Dose 1 | 0.093469 | - |
|  | Dose 2 | 2.9e-05 | *** |
|  | Dose 3 | 0.000187 | *** |
| scan | Dose 1 | 0.439412 | - |
|  | Dose 2 | 0.0002445 | *** |
|  | Dose 3 | 0.0002445 | *** |


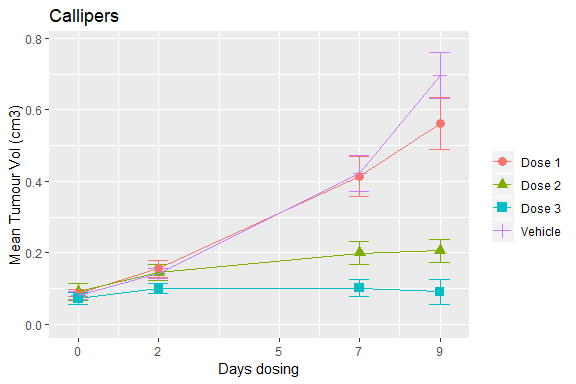


Figure G: Calliper tumour volume for an AZ study on 39 female Balb/c mice with the CT26 tumour. We display the mean and standard error of mice in the same group across time.


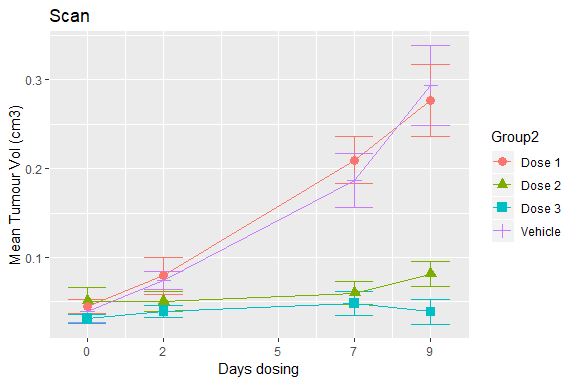


Figure H: Scan tumour volume for the same study as in Figure D (AZ study on 39 female Balb/c mice with the CT26 tumour). We display the mean and standard error of mice in the same group across time.


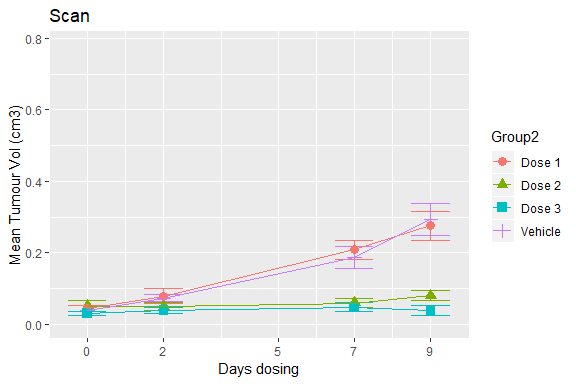


Figure I: Scan tumour volume for the same study as in Figure G (AstraZeneca’s study on 39 female Balb/c mice with the CT26 tumour). We display the mean and standard error of mice in the same group across time. Y-axis is scaled to match Figure G.

## Further details on Inter-Operator variability

Complementing the analysis presented in the main text, where we detailed inter-operator precision in volume estimates for BioVolume and callipers, we present here the inter-operator precision for length, width and eight measurements made using BioVolume. We also show precision values for BioVolume’s estimates of tumour area and volume for three volume formulae (spheroid, ellipsoid and cylindrical). For length and width, the agreement between two different operators was very high, which indicates that the thermal signature of tumours is a very reliable means of delineating the boundary of a tumour. The consistency of these measurements is also reflected in the tumour area (Figure J). By visual inspection of the plots, the segmentation is not very sensitive to natural rotation of the mouse. Height was the most variable of the linear measurements. This is due to partly the fitting of the back of the mouse, the calculation of the normal and the calculation of the top height. This part of the algorithm will be refined in future versions. Given the high consistency of length and width measurements relative to height, it follows that the spheroid formula is very precise, whereas the ellipsoid and cylindrical formulae, which incorporate height, are more variable.


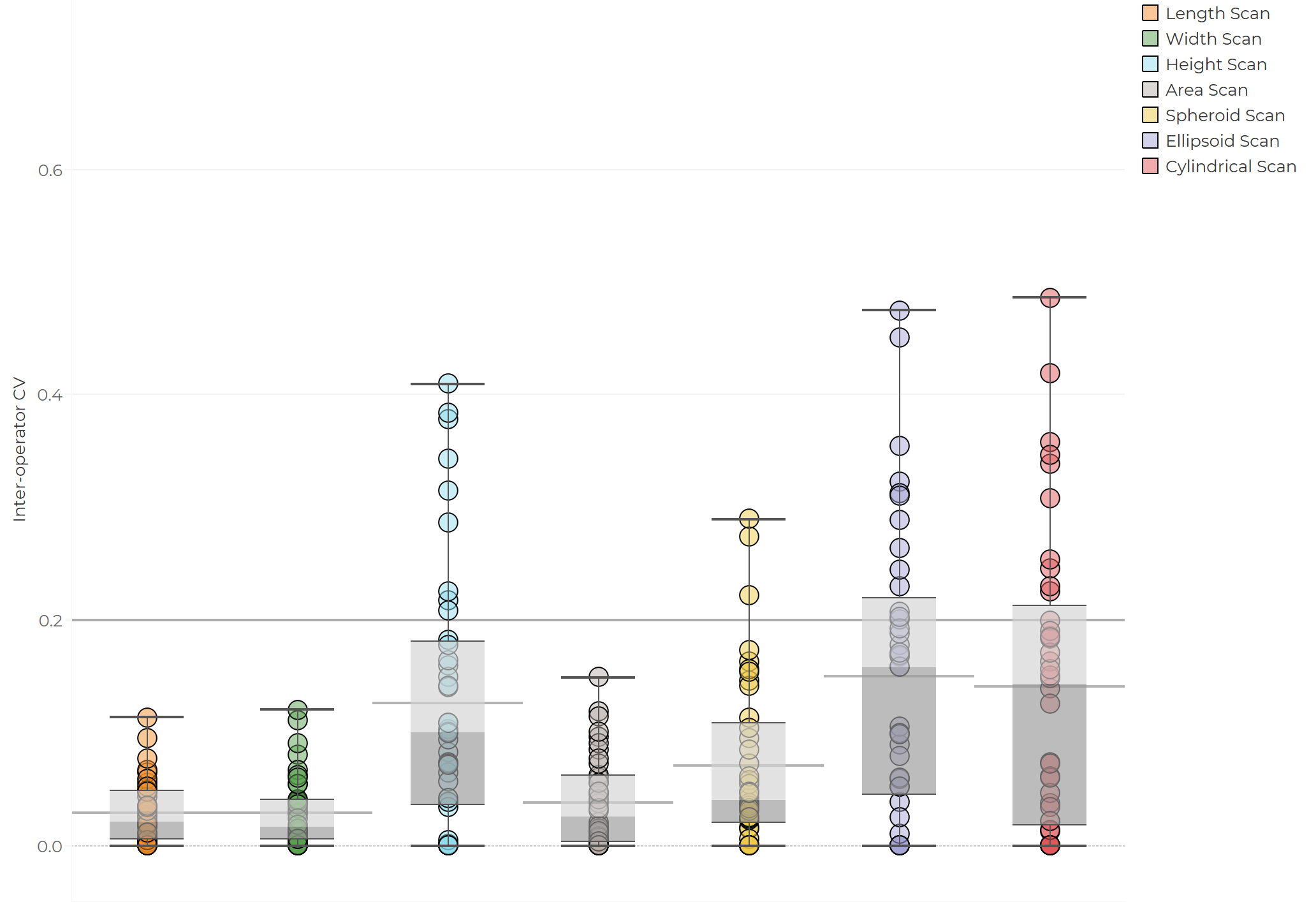


Figure J: Inter-operator CV for linear dimensions, area and volumes for BioVolume

The numbers of repeats/operators used to construct Figure 9 and Figure J from Dataset 2 were two in almost 50% of the cases, 25% of them contained three repeats and 25% four or more (Figure K).


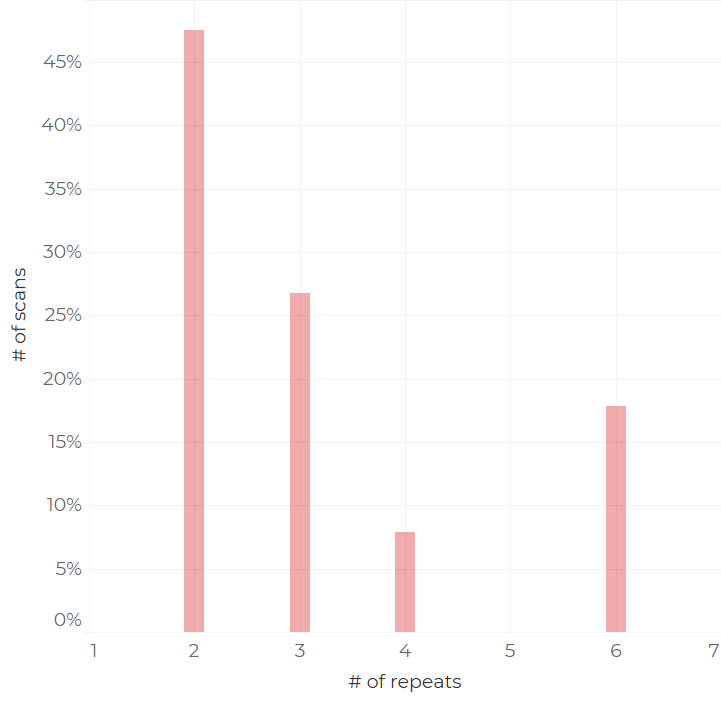


Figure K: number of scans versus number of repeats used in the inter-operator CV with filters applied.

## A note on power analysis

We performed a simple analysis of variance analysis based on the coefficient of variation (CV). As explained in the methods section, this is a powerful metric that is directly linked to a two-sample ANOVA test which is widely used for power calculations and study design. Overall, 73% of measures were below the acceptance threshold of 0.2 with most of the contributors coming from the Balb/C group (see Figure L).


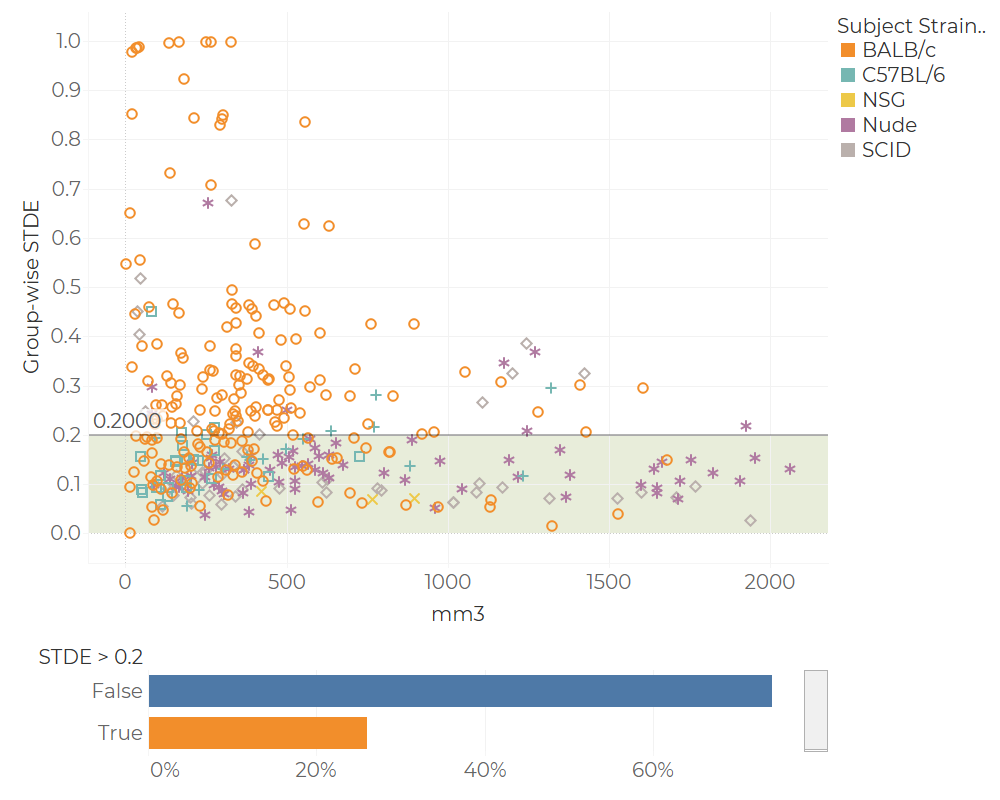


Figure L: Summary of standard error (STDE) in volume by mouse strain. 22% of the mice overall show a coefficient of variation larger than 0.2 with the highest incidence in the Balb/C group (56%). Excluding Balb/C only 13% lie above the threshold.
