# Supplementary material for: An innovative non-invasive technique for subcutaneous tumour measurements
Source: PLoS One. 2019 Oct 14;14(10):e0216690. doi: 10.1371/journal.pone.0216690 (PMC6791540; doi:10.1371/journal.pone.0216690)
Supplement: S2 Appendix — (DOCX) [file pone.0216690.s002.docx]

**S2 Appendix - Supplementary materials of Tumour Growth Modelling**

**From paper:**

Delgado-SanMartin et al. An innovative non-invasive technique for subcutaneous tumour measurement, submitted to PLOS ONE, 2019

# Background

Mathematical models have been long used in cancer research to describe, explain and predict tumour growth and treatment efficacy [1]. A major advantage of mathematical models in cancer research is that they may be applied to rapidly test hypotheses pertaining to tumour growth and treatment. Here, we use a 3-dimensional cellular automaton (CA) model to simulate the growth and treatment of subcutaneous tumours and to assess the discrepancy between tumour volumes estimated with archetypical callipers and the actual tumour volumes. We place particular emphasis on the accuracy of treatment efficacy metrics.

# Methods

## Basic stochastic cellular automaton model

The basic 3D stochastic CA model is designed to replicate the growth of subcutaneous tumours in laboratory animals. The model is implemented on a regular 3D grid divided into a number of equally sized cubes. As we assume a constant density of cancer cells throughout the tumour (*ρ* = 4.0×10^8^ cells/cm^3^) each non-empty cube in this 3D space is assumed to contain *N_C_* = 400 cancer cells and has a size (the length of a side of each cube) of *l* = 100 µm. This “many to one” approach allows us to simulate the growth of a large number of tumours with realistic sizes within a reasonable amount of time.

Unless otherwise stated, we consider two populations of cells within each tumour: i) population 1—the primary cell population implanted under the animal’s skin; ii) population 2— a secondary, more aggressive cell population arising during tumour growth from spontaneous mutations. All simulations begin with only population 1 cells present. However, during cell division, population 1 cells can mutate into population 2 cells with probability *p_mut_* = 0.001.

Model simulations begin by placing a cluster of population 1 cells in the centre of the bottom of the computational domain which replicates the process of injecting cancer cells under the skin of a laboratory animal. We impose no-flux Neumann boundary conditions on the domain which reflects the assumptions that cancer cells are not able to penetrate the animal’s body.

The model’s state updates at discrete time intervals of length *τ* = 12 hours and all models are simulated for *t* = 30 days. At each time interval population 1 cells residing in the cubes that have at least one unoccupied neighbour in the first order Moore neighbourhood^[[1]](#footnote-1)^ divide with probability *p_div1_* = 0.7. Population 2 cells divide with probability *p_div2_* = 0.9. The direction of division is random. Cells that do not have any free neighbours are assumed to be quiescent (they do not divide). The height of the *in-silico* tumours is regulated by a *bias* parameter that penalises cells for dividing upwards (in the positive z-direction). Thus, the greater the value of *bias,* the lower the probability that the cells divide upwards. Unless otherwise stated, we set *bias* = 0.9.

## Tumour morphologies

To generate *in silico* tumours with different morphologies, representative of those observed in the laboratory, we implement a number of different modifications to the basic CA model. We emphasise at this point that our objective was not to accurately reproduce the growth mechanisms of subcutaneous tumours with different morphologies but rather, to capture their growth kinetics. Below, we describe the modifications that enable us to achieve this objective.

### Tumours with one, two and three peaks

The only difference between tumours with one, two, and three peaks are the initial conditions. Single peak tumours are generated by placing one semi-spherical cluster of cells with *radius = 0.3* cm in the centre of the bottom of the lattice. Double and triple peak tumours are generated by replacing the single cluster of cells with two and three semi-spherical clusters with radii equal to 0.24 cm and 0.21 cm respectively.

### Igloo

The igloo-like tumour morphology is achieved by adding a 0.60 cm by 0.01 cm by 0.01 cm tail of cells to a single semi-spherical cluster with *radius* = 0.3 cm as the initial condition.

### Birthday cake

The birthday cake morphology is characterised by a secondary tumour growing out of the primary one. We generated these tumours by introducing another population of cells—population 3—that divides with probability *p_div3_* = 1. At time *t* = 0, a randomly selected single cube at the tumour’s surface is filled with cells from population 3 rather than with cells from population 1, thus establishing a very aggressive clone that leads to a layered morphology.

### Volcano

Generating tumours with hollow centres is difficult to achieve without introducing additional assumptions. In particular it cannot be done by manipulating the initial conditions alone. Since the actual mechanisms governing the growth of tumours are not of utmost importance to the present study, we introduced a 2D Gaussian function to manipulate the probability of cancer cell division based on the location of the cells on the xy-plane cross-section taken through the computational domain. Thus the probability of division of population 1 cells at location *(x,y,•)* is given by *p_div1_ × f(x,y)* where *f(x,y)* is the 2D Gaussian function visualised in *Figure S2.1*. Similarly, the probability of division of population 2 cells at location *(x,y,•)* is given by *p_div2_ × f(x,y)*. Consequently, cells residing closer to the centre of the xy-plane of the domain divide with lower probability that cells further away from the centre.


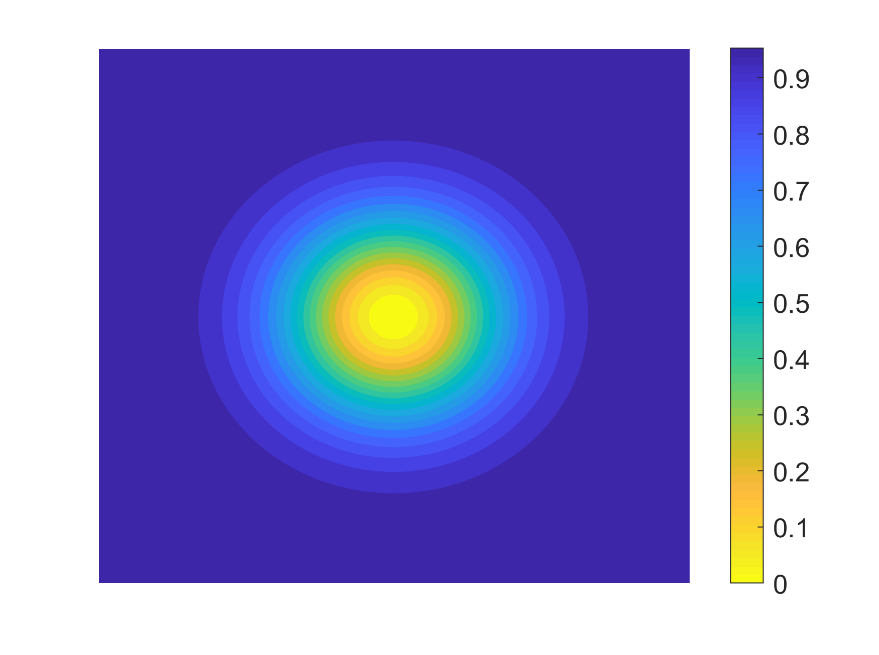


Figure S2.1: 2D Gaussian function describing the probability of division of cancer cells depending on their location in the cross section of a volcano tumour in the xy-plane.

## Tumour treatment

In addition to growth, we also simulated treatment of the *in-silico* tumours. Treatment was applied between days 15 and 25. Since anticancer treatments, such as radio or chemotherapy, typically target actively proliferating cells we assume that only cells on the surface of *in silico* tumours are affected by treatment. Treated tumours are subject to lower proliferation rates and cell death. Specifically, the division probability of cells from population *i* during the treatment period is given by *p_tdivi_ =* *p_divi_ × λ* where *λ = 0.5* and the probability of death is given by *p_remi_ = p_tdivi_ × (1 – λ).* Dead cells are simply removed from the computational domain. A schema of the four model parameters is shown in *Figure S2.2*

*Figure S2.2: Schema of the model parameters. Cells are represented by isometric square voxels.*

## Tumour volume measurements

We measured volumes of our *in-silico* tumours at 3-day time intervals until *t =* 30 days. Two volumes were derived for each tumour: i) the ground truth volume (GT) and ii) the ellipsoidal volume estimated from simulated calliper (SC) measurements. Although in reality it is very difficult to accurately measure tumour volume, one of the main advantages of using *in silico* tumours is that the volume can be determined exactly. Since we know the volume of each cube occupied by cancer cells (*V_cube_* = 1.0 × 10^-6^ cm^3^) we can calculate the ground truth (GT) volume of *in silico* tumours using the formula *V_GT_(t)* = *M(t)* × *V_cube_* where *M(t)* is the number of cubes occupied by cancer cells at time *t*.

We simulated canonical calliper measurement by identifying the two longest perpendicular lines connecting any four points across every xy-plane within the tumour. We defined the longer line as tumour length *L(t)* and the shorter line as tumour width *W(t)* and then estimated the volume by using the spheroid formula *V_SC_(t) = 0.5* × *L(t)* × *W(t)^2^*.

## Tumour Growth Inhibition and Area Under the Curve

The Tumour Growth Inhibition (TGI) index is a commonly used metric for the assessment of treatment efficacy in preclinical trials. We used the following formula to determine the TGI index:

$TGI=\left( 1-\frac{V_{T}\left( t \right) V_{C}\left( 0 \right)}{V_{T}\left( 0 \right) V_{C}\left( t \right)} \right)\times100\%$

where *V_T_(t)* and *V_C_(t)* are the volumes of treated and control tumours at time *t* and *V_T_(0)* and *V_C_(0)* are the volumes of treated and control tumours at time 0, respectively.

The TGI index uses only a single time point to assess treatment efficacy, ignoring the growth kinetics of the tumour. Hence, we also computed the Area Under the Curve (AUC) index, which integrates information from the entire growth curve. We calculated AUC using the formula:

$AUC=\left( 1-\frac{{AUC}_{T}}{{AUC}_{C}} \right)\times100\%$

where *AUC_T_* and *AUC_C_* are the areas under the treated and control growth curves, respectively. The AUC was calculated using MATLAB’s *trapz* function.

# Results

We interrogated the model with the objective to compare the impact that different tumour morphologies would have in the assessment of treatment efficacy using two metrics: Callipers and Ground Truth (see *Figure S2.3*).


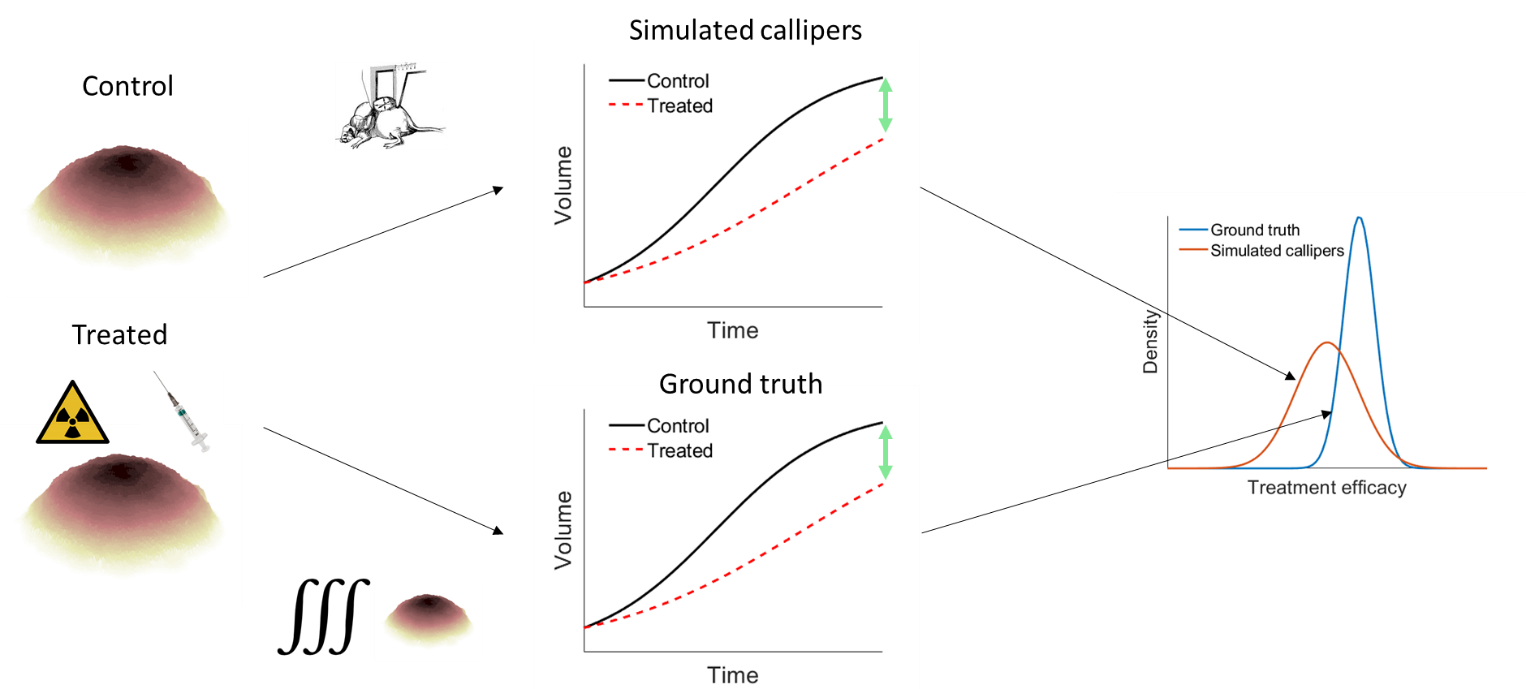


*Figure S2.3: Schema of the in-silico schedule.*

As shown in Figure 5 of the main text, tumour volumes calculated using simulated calliper measurements significantly overestimated tumour volume. However, a scientist conducting preclinical trials is more likely to be interested in the relative difference between the control and treated groups than in the actual volumes. Therefore, we used two metrics to quantify the treatment efficacy when tumour volume is estimated using SC measurements and compared them to the actual effect on the GT volume.

A commonly used measure of treatment efficacy is the Tumour Growth Inhibition (TGI) index. We calculated the TGI for all six morphologies using days 18, 24 and 30 as the study end-points. Figure 6 shows histograms of the TGI computed using the GT volumes (shades of blue) and the volumes estimated using SC measurements (shades of red). The index was calculated for every pair of control and treated growth curves for each morphology.

The histograms of SC-estimated volumes are wider than those of the GT volumes with significantly larger standard deviations (Table S2.1). The TGI for the GT volume tends to be the lowest when day 18 is used as the end-point, largest for day 24 and lie in between when measured on day 30 (Figure 6; blue histograms). This reflects the fact that the treatment was applied between days 15—25 and hence the largest effect is observed just before the end of the treatment (see Figure 6). On the other hand, the TGI histograms for SC-estimated volumes were very similar when either day 24 or 30 are used as the end-points (Figure 6; red histograms). This could be attributed to the growth dynamics of treated tumours being insufficiently captured with callipers.

Comparing the TGI index between the SC and GT volumes reveals that SC estimated volumes heavily underestimate the effects of treatment when days 18 and 24 were used as the study end-points (Figure 5). Although the differences were less pronounced when using day 30 as the end-point (the 95% confidence intervals overlap for single peak, double peak, triple peak and birthday cake tumours; Table S2.1), there is statistically significant evidence that the TGI indices differ between the two measurements methods (Table S2.2).

Since the TGI index calculations use only a single time point we also computed the Area Under the Curve (AUC) index which incorporates the dynamics of the entire growth curve. Although the SC-estimated AUC values were more variable than those calculated for the GT volumes, the variability was less prominent for tumours with double and triple peak morphologies (Figure S2.3 and Table S2.3). Recall that to generate double and triple peak tumours we seeded two and three clusters of cells at *t* = 0, respectively. Although the initial clusters had fixed radii for both types of tumour their location was randomised. Thus, in some cases the clusters overlapped resulting in lower initial volume. This is illustrated in Figure S2.4.


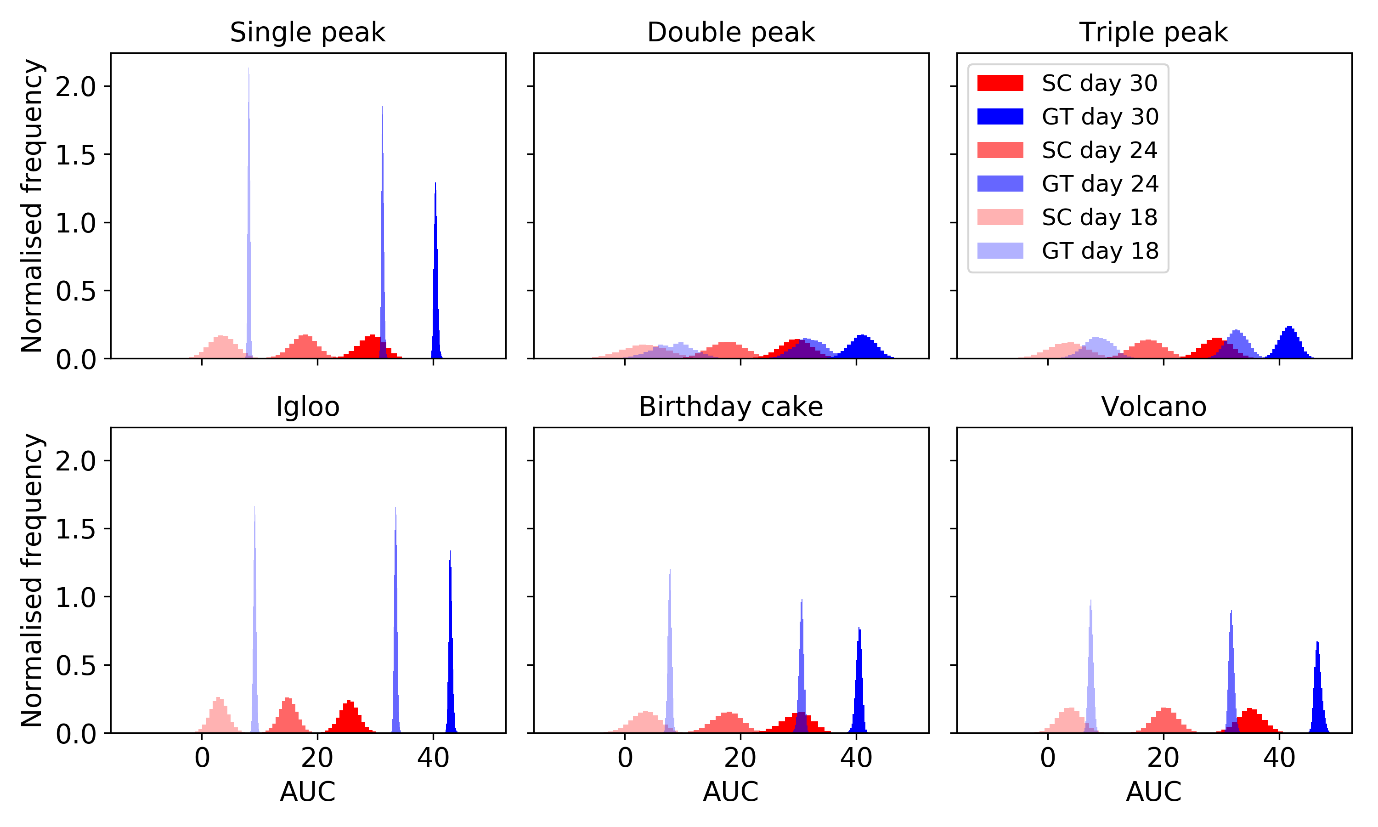


Figure S2.4: Histograms showing the Area Under Curve (AUC) index computed for different morphologies using the true volume (shades of blue) and simulated calliper measurements (shades of red). The AUC was computed using days 18, 24 and 30 as experiment end-points.

Figure S2.5A shows the initial clusters seeded sufficiently away from each other that they do not overlap much Conversely, the clusters in Figure S2.5B overlap substantially such that the volume of the tumour in Figure S2.5B is smaller than the volume of the tumour in Figure S2.5A. This variability in the initial conditions leads to higher variability observed in the AUC estimates.


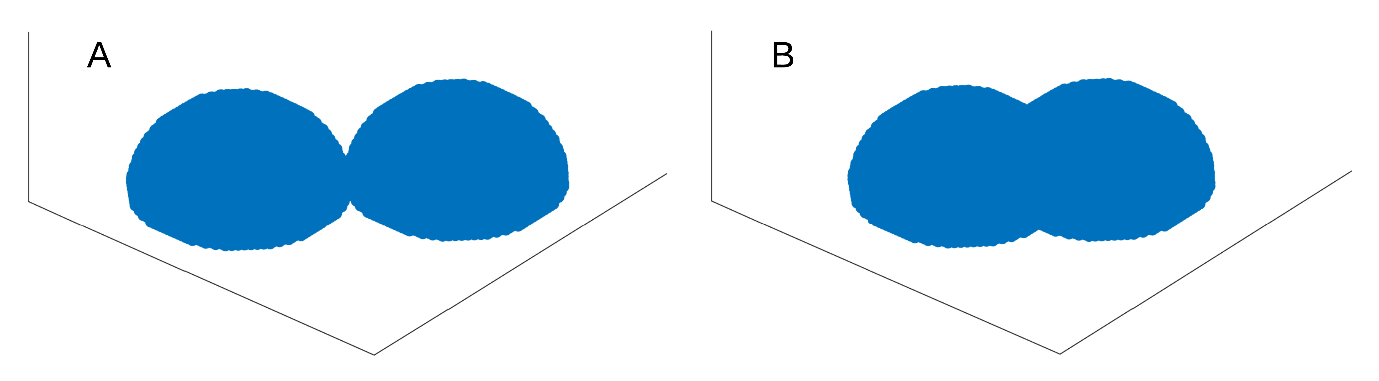


Figure S2.5: Initial seeds of double peak tumours. A - the two clusters are seeded apart from each other so they barely overlap; B - the clusters are seeded close to each other and overlap substantially.

The results of the AUC index calculations were more consistent than those for the TGI in that increasing the study end-point results in higher values of the index for both SC and GT volumes (Figure S2.4). Although calliper-estimated volumes consistently led to lower AUC values, the SC-estimated AUC indices are statistically significantly different from their GT equivalents regardless of the study end-point and tumour morphology as assessed by the paired t-test (Table S2.4).

Finally, we combined the results of all the morphologies to create the distributions shown on Figure S2.6. Although the distributions for the TGI and the AUC for both SC and GT volumes overlap, the distributions for the SC volumes are markedly wider and their central values differ from those computed for the GT volumes (Figure S2.6, and Tables S2.1-S2.6).

Figure S2.6: Histograms showing the TGI (A) and AUC (B) indices computed across all morphologies for the true volume (blue) and simulated calliper measurements (red) using days 18, 24 and 30 as the experiment end-points. Since we generated 10^3^ growth curves for each morphology (control and treated) each histogram is composed of 3.6×10^3^ data points.

Table S2.1: Summary statistics for the TGI histograms from Figure S2.6 (day 30).

|  | Measurement method | Mean | Median | Standard deviation | 95% confidence intervals | Interquartile range |
| --- | --- | --- | --- | --- | --- | --- |
| Single peak | SC | 44.03 | 44.22 | 3.92 | (35.56, 51.16) | (41.64, 46.68) |
|  | GT | 48.22 | 48.20 | 0.54 | (47.21, 49.32) | (47.84, 48.58) |
| Double peak | SC | 44.78 | 44.85 | 2.78 | (39.12, 50.06) | (42.95, 46.68) |
|  | GT | 48.81 | 48.81 | 0.67 | (47.45, 50.15) | (48.38, 49.24) |
| Triple peak | SC | 44.33 | 44.43 | 2.48 | (39.21, 48.90) | (42.71, 46.06) |
|  | GT | 48.99 | 49.00 | 1.08 | (46.85, 51.07) | (48.25, 49.74) |
| Igloo | SC | 40.87 | 40.92 | 2.54 | (35.69, 45.72) | (39.23, 42.58) |
|  | GT | 50.40 | 50.38 | 0.49 | (49.49, 51.40) | (50.07, 50.72) |
| Birthday cake | SC | 45.12 | 45.43 | 3.95 | (36.43, 52.06) | (42.73, 47.84) |
|  | GT | 49.83 | 49.88 | 0.79 | (48.13, 51.23) | (49.33, 50.38) |
| Volcano | SC | 52.09 | 52.31 | 3.31 | (44.87, 58.04) | (50.08, 54.35) |
|  | GT | 61.72 | 61.68 | 0.83 | (60.18, 63.46) | (61.14, 62.25) |

Table S2.2: Mean t-scores ± 95% confidence intervals obtained in the paired t-test for the difference between TGI indices obtained for the two measurement methods. The critical value of t is 1.96.

| day | Single peak | Double peak | Triple peak | Igloo | Birthday cake | Volcano |
| --- | --- | --- | --- | --- | --- | --- |
| 18 | 7027.71±0.01 | 6568.65±0.01 | 7284.14±0.01 | 10249.64±0.01 | 4359.95±0.01 | 4738.20±0.01 |
| 24 | 10305.81±0.01 | 9982.52±0.01 | 10837.78±0.01 | 13627.32±0.01 | 6878.75±0.01 | 6999.46±0.01 |
| 30 | 1218.22±0.01 | 1337.81±0.01 | 1562.28±0.01 | 3795.57±0.01 | 1170.03±0.01 | 3016.47±0.01 |

Table S2.3: Summary statistics for the AUC histograms from Fig. S2.3 (day 30).

|  | Measurement method | Mean | Median | Standard deviation | 95% confidence intervals | Interquartile range |
| --- | --- | --- | --- | --- | --- | --- |
| Single peak | SC | 29.23 | 29.31 | 2.32 | (24.42, 33.55) | (27.75, 30.82) |
|  | GT | 40.41 | 40.4 | 0.31 | (39.86, 41.05) | (40.2, 40.61) |
| Double peak | SC | 29.53 | 29.57 | 2.73 | (24.09, 34.73) | (39.51, 42.55) |
|  | GT | 41.04 | 41.05 | 2.20 | (36.66, 45.18) | (39.51, 42.55) |
| Triple peak | SC | 29.08 | 29.12 | 2.55 | (23.98, 33.95) | (27.38, 30.82) |
|  | GT | 41.63 | 41.64 | 1.60 | (38.48, 44.71) | (40.53, 42.75) |
| Igloo | SC | 25.47 | 25.48 | 1.69 | (22.1, 28.79) | (24.37, 26.59) |
|  | GT | 42.97 | 42.95 | 0.31 | (42.41, 43.6) | (42.76, 43.16) |
| Birthday cake | SC | 29.66 | 29.84 | 2.65 | (24.02, 34.38 | (27.99, 31.5) |
|  | GT | 40.42 | 40.45 | 0.53 | (39.3, 41.34 | (40.09, 40.78) |
| Volcano | SC | 34.96 | 35.01 | 2.26 | (30.35, 39.29) | (33.51, 36.47) |
|  | GT | 46.68 | 46.64 | 0.61 | (45.61, 48.0) | (46.25, 47.05) |

Table S2.4: Mean t-scores ± 95% confidence intervals obtained in the paired t-test for the difference between AUC indices obtained for the two measurement methods. The critical value of t is 1.96.

| Experiment end-point (day) | Single peak | Double peak | Triple peak | Igloo | Birthday cake | Volcano |
| --- | --- | --- | --- | --- | --- | --- |
| 18 | 1271.61±0.01 | 3335.04±0.01 | 3410.73±0.01 | 4120.42±0.01 | 1710.31±0.01 | 1798.32±0.01 |
| 24 | 4376.99±0.01 | 9311.03±0.01 | 9229.33±0.01 | 12264.62±0.0 | 5042.65±0.01 | 5433.15±0.0 |
| 30 | 3960.75±0.01 | 6856.84±0.01 | 7262.95±0.01 | 10494.40±0.0 | 4003.13±0.01 | 5467.13±0.01 |

Table S2.5: Summary statistics for the TGI histograms from Figure 6 of the main text

| Experiment end-point (day) | Measurement method | Mean | Median | Standard deviation | 95% confidence intervals | Interquartile range |
| --- | --- | --- | --- | --- | --- | --- |
| 18 | SC | 44.79 | 44.94 | 8.14 | (27.61, 60.03) | (40.27, 50.42) |
|  | GT | 50.85 | 49.01 | 8.01 | (38.20, 68.45) | (47.66, 51.82) |
| 24 | SC | 44.50 | 45.90 | 10.18 | (20.15, 60.46) | (40.40, 51.02) |
|  | GT | 50.99 | 49.14 | 7.28 | (40.92, 67.15) | (46.89, 53.80) |
| 30 | SC | 42.54 | 45.12 | 18.83 | (-5.67, 69.22) | (30.92, 57.02) |
|  | GT | 51.14 | 51.53 | 6.70 | (37.04, 61.90) | (46.81, 55.99) |

Table S2.6: Summary statistics for the AUC histograms from Figure 6 of the main text.

| Experiment end-point (day) | Measurement method | Mean | Median | Standard deviation | 95% confidence intervals | IQR |
| --- | --- | --- | --- | --- | --- | --- |
| 18 | SC | 31.30 | 33.70 | 20.96 | (-18.83, 62.14) | (20.82, 45.99) |
|  | GT | 49.22 | 49.49 | 6.73 | (35.19, 60.83) | (44.04, 53.98) |
| 24 | SC | 27.78 | 30.66 | 22.36 | (-26.26, 60.61) | (17.22, 43.45) |
|  | GT | 44.06 | 44.86 | 7.60 | (28.78, 57.40) | (38.19, 49.75) |
| 30 | SC | 26.3 | 29.47 | 23.28 | (-30.02, 60.18) | (15.52, 42.33) |
|  | GT | 41.74 | 42.61 | 7.68 | (26.27, 55.33) | (35.79, 47.47) |

## Parameter sensitivity analysis

To test the robustness of the model results, and to identify potential relationships between the parameters and outputs we conducted a sensitivity analysis of the model reproducing the single peak morphology. Recall that the parameter *bias* penalises cells for dividing in the positive z-direction. If *bias* = 0 the cells are equally likely to divide in any direction whereas if *bias* = 1 the cells can only divide in the xy-plane and/or negative z-direction. We introduced the *bias* parameter to account for the fact that cancer cells tend to maximise their contact surface with the host to maintain access to nutrients [2]. Also, recall that *p_div1_* represents the probability of division for population 1 cells in the absence of treatment while *λ* can be thought of as a treatment factor with values in the range (0,1). Low values of *λ* correspond to a high treatment effect, i.e., the probability of division of cells during treatment is relatively low and their probability of death is relatively high. On the other hand, high values of *λ* coincide with a low treatment effect. Additionally, let us define by *λ_len_* the duration (in days) of a generic anticancer treatment that begins at time *t* = 15 [days]. Thus, *λ_len_* can take values in the range (0,15).

We studied the impact of varying the parameters *bias*, *p_div1_*, *λ* and *λ_len_* on the model outputs. We simulated the model 2.5 × 10^3^ times by sampling values for each of the four parameters from uniform distributions *U*. Specifically, we sampled from: *bias ~ U*(0, 1), *p_div1_* ~ *U*(0.1, 0.9), *λ* ~ *U*(0.1, 0.9), *λ_len_* ~ *U*(0, 15). For each set of sampled parameter values, we simulated the growth of control and treated tumours six times and computed the TGI and AUC indices for both GT and SC volumes.

Figure S2.7 shows the relationships between the model parameters and tumour height when measured at *t* = 30 days. We also computed the linear correlation coefficient *r*. Although *r* = -0.83 for the *bias*-*height* pair, the relationship is clearly nonlinear as assessed by the scatter plot. As *bias* increases the *height* decreases. The decrease is initially linear but as *bias* increases beyond approximately 0.9, the value of *height* drops rapidly revealing a logarithmic relationship. Since *bias* penalises cells for proliferating in the positive z-direction it is not surprising that the *height* decreases with growing *bias*.

The probability of division by population 1 cells *p_div1_* appears to have a weak positive correlation with *height* (*r* = 0.35). As *p_div1_* increases the tumour volume at *t* = 30 also increases (*r* = 0.69, data not shown). This is because there are simply more cells that can divide in the positive z-direction for a given value of *bias*. Parameters *λ* and *λ_len_* are not correlated with *height*.


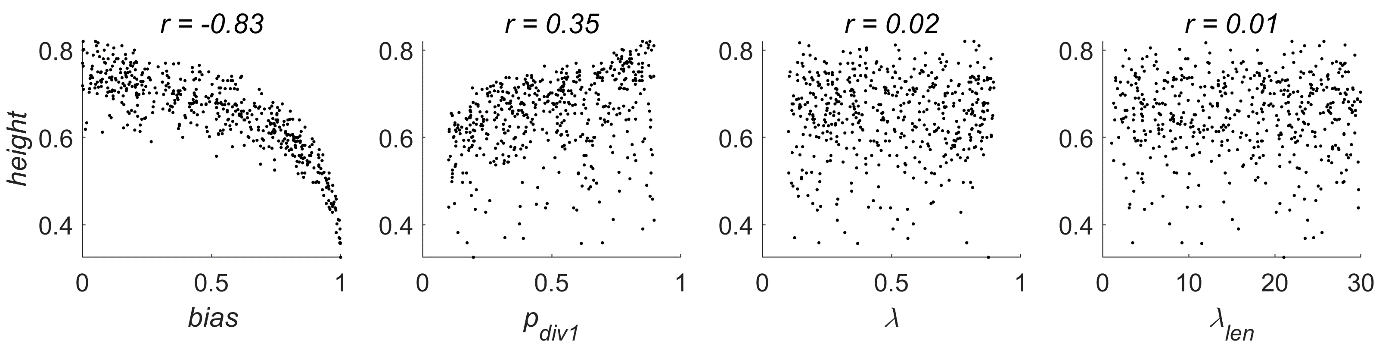


Figure S2.7: Correlations between selected model parameters and the tumour height measured at t = 30.

Next, we assessed the relationships between the model parameters and the values of the TGI and AUC indices for the GT volumes. There was no correlation between *bias* and *p_div1_* for either of the indices (Figure S2.8). However, there were obvious relationships for *λ* and *λ_len_*. Since greater values of *λ* correspond to lesser treatment effects it is not surprising that *λ* was negatively correlated with the TGI and AUC (*r* = -0.75 and *r* = -0.77, respectively). Similarly, increasing the treatment duration appears to coincide with greater treatment effect. Thus *r* = 0.57 and *r* = 0.52 for the *λ_len_*-TGI_GT_ *λ_len_*-AUC_GT_ pairs, respectively. Similar trends were observed for the volumes obtained with SC measurements (data not shown).


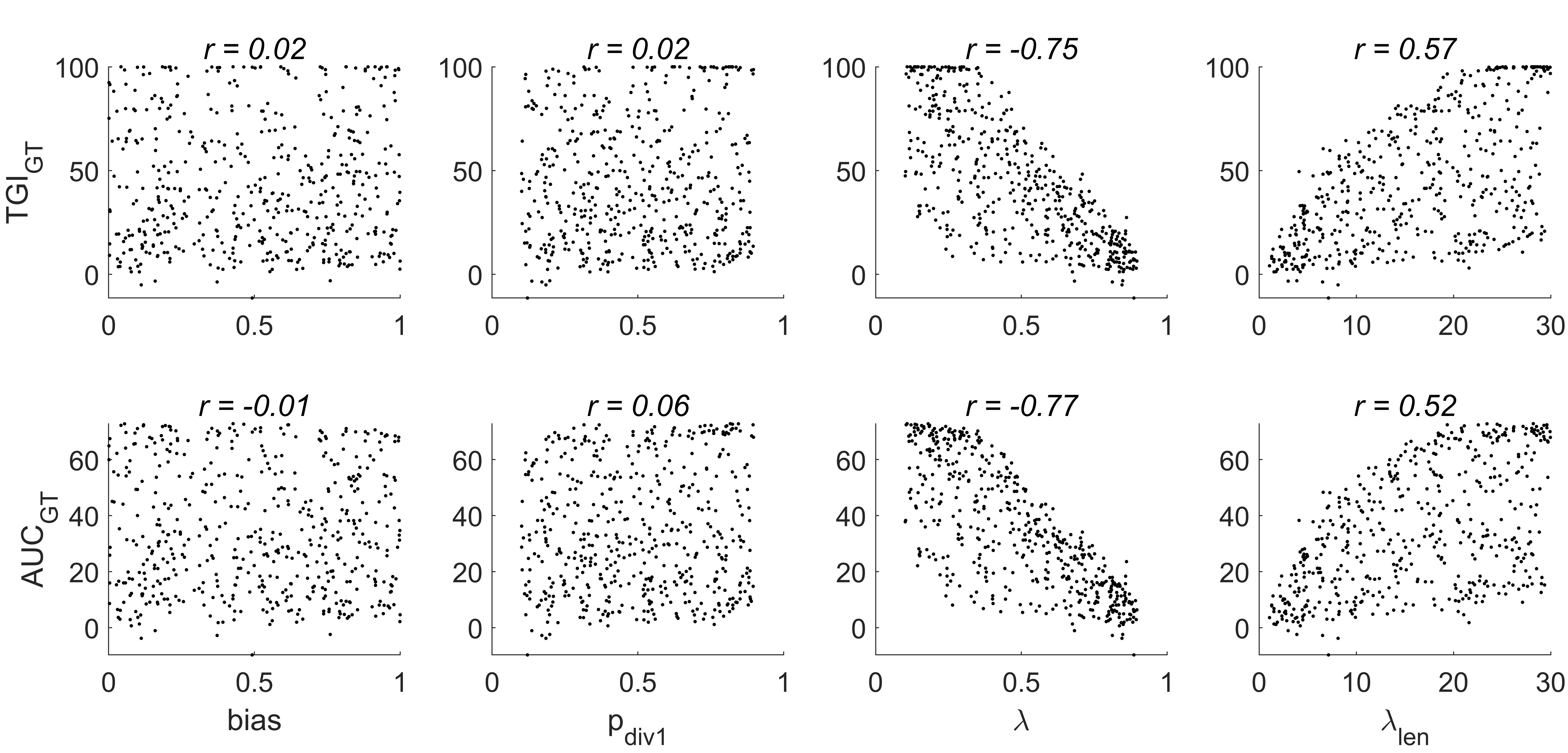


Figure S2.8: Correlations between selected model parameters and the values of TGI and AUC indices calculated using the ground truth volumes.

The similar relationships between *λ* and *λ_len_* and the TGI and AUC indices observed in Figure S2.8 prompted us to examine the link between TGI and AUC indices. As shown in Figure S2.9, the values of TGI and AUC exhibit nearly perfect linear relationship for both GT and SC measurement methods, with the variance increasing with treatment effect potency.

Figure S2.9: Relationships between the TGI and AUC indices for both GT and SC volumes.


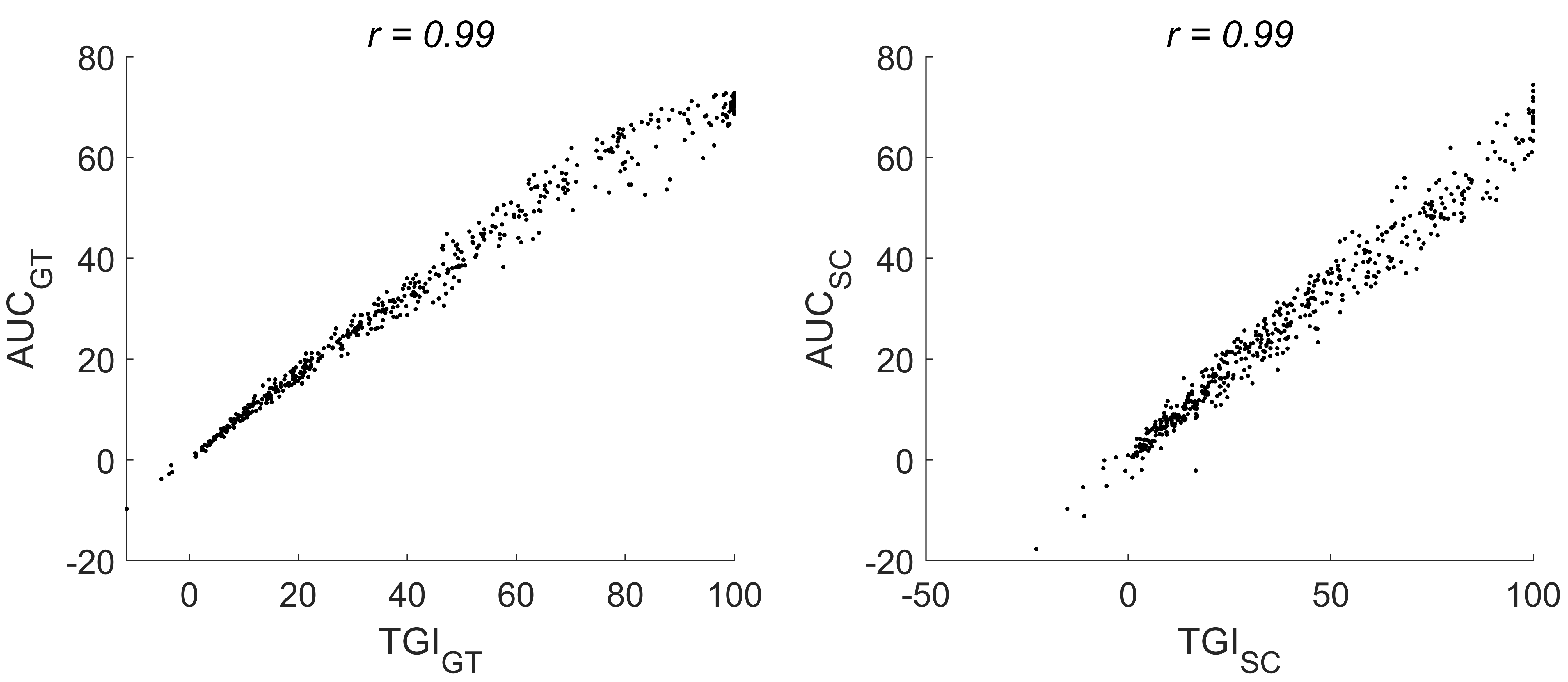


As the main objective of this model is to investigate the impact of estimating tumour volume from calliper measurements on the accuracy of treatment efficacy metrics, we also explored the effect of varying model parameters on the discrepancy between TGI and AUC indices for the GT and SC measurement methods. Figure S2.10 reveals that neither *bias*, *p_div1_* nor *λ* are correlated to the differences between TGI and AUC for tumours measured with GT and SC-estimated volumes. However, we observed weak linear relationships for the *λ_len_* parameter (*r* = 0.31 and *r* = 0.51 for the differences in TGI and AUC, respectively) which indicates that the longer the treatment duration, the more difficult it is to estimate the TGI and AUC indices. This observation is consistent with the scatter plots for *λ_len_* in Figure S2.8.

Figure S2.10: Relationships between model parameters and the differences in TGI and AUC indices computed with GT and SC-estimated volumes.


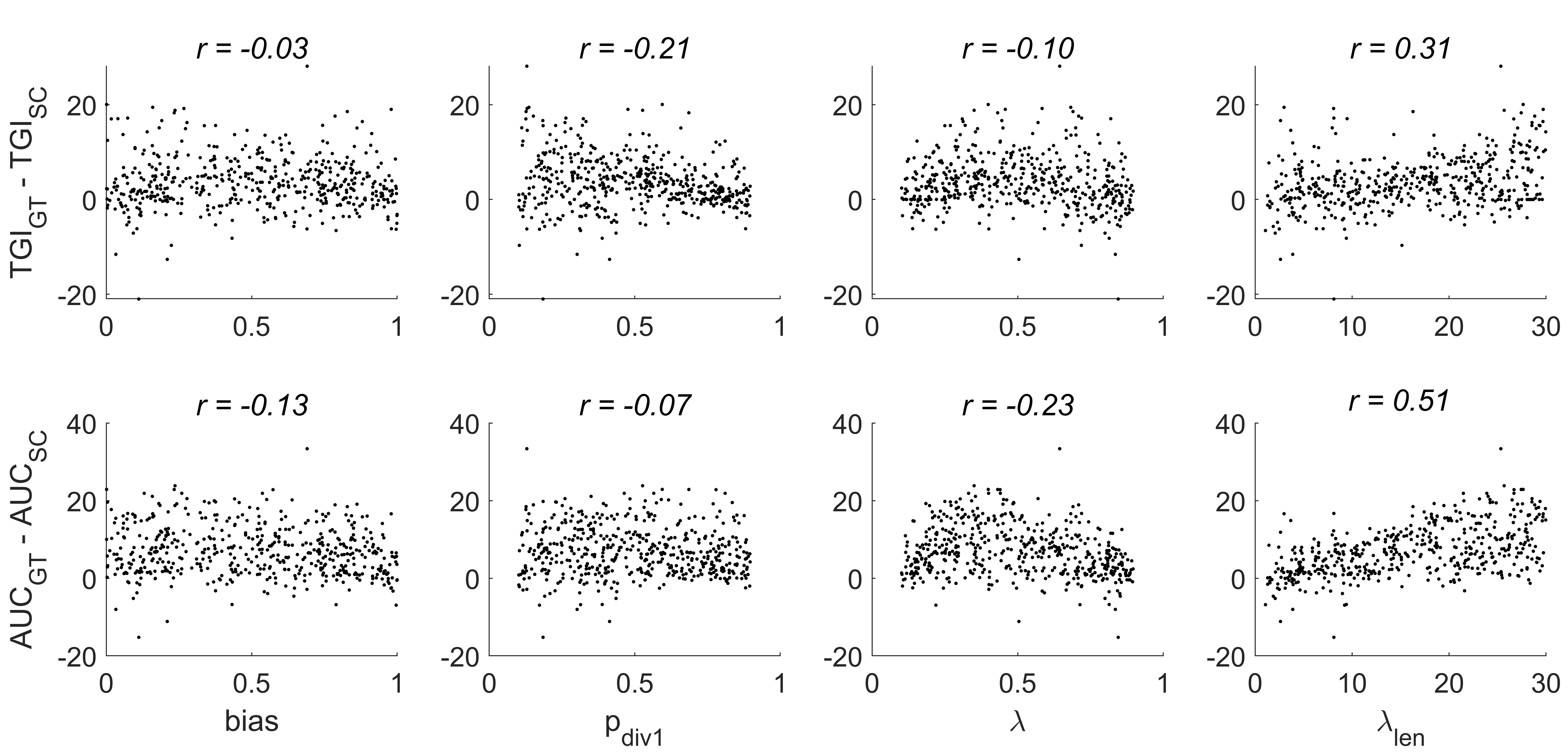


Finally, we compared the TGI and AUC values obtained for the GT and SC volumes. Figure S2.11 shows a very strong linear correlation for the TGI index (*r* = 0.98). The red lines represent a theoretical perfect 1:1 match between the values. Thus, using SC-estimated volumes generally results in underestimating the TGI and AUC indices. Although *r* = 0.96, the relationship between AUC_GT_ and AUC­_SC_ appears to be nonlinear for higher values of AUC.


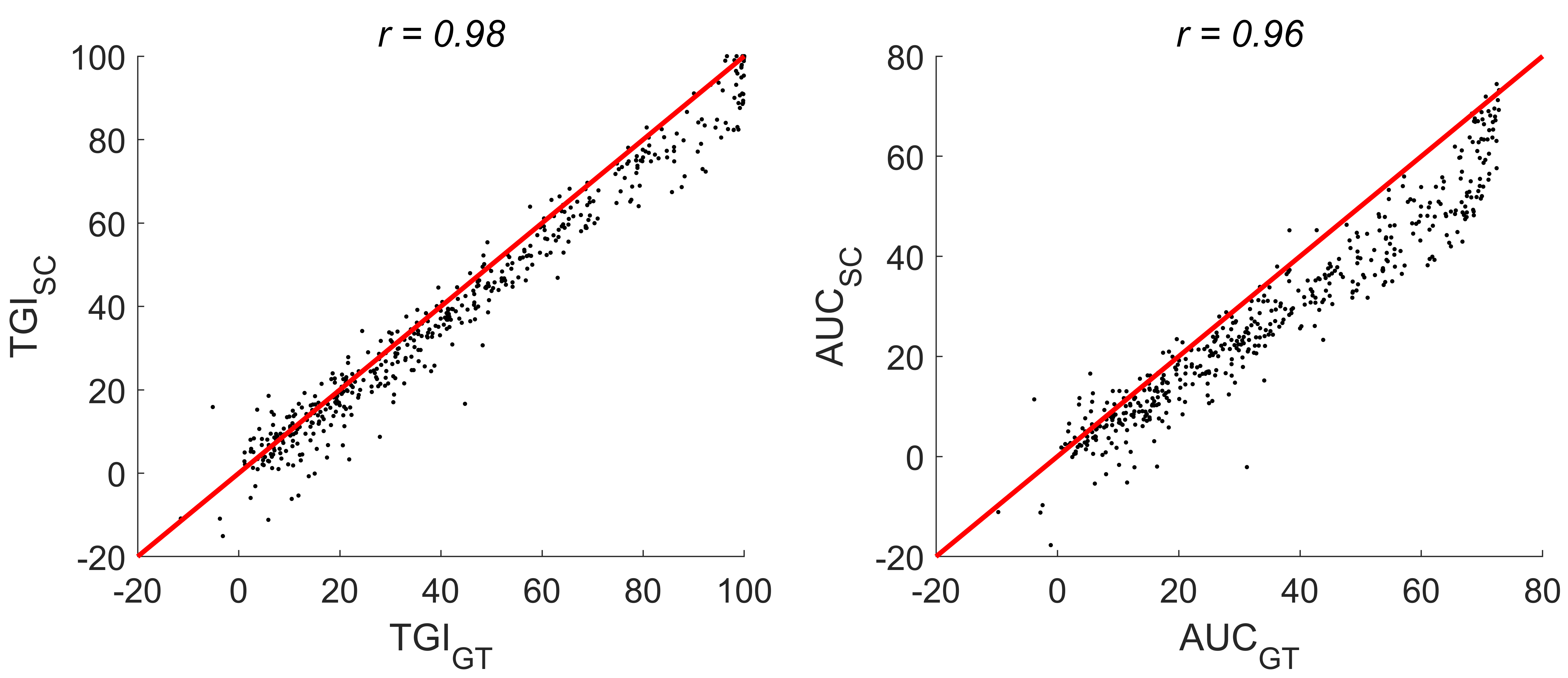


Figure S2.11: Correlations between the TGI and AUC indices calculated with GT and SC-estimated volumes. Red lines indicate a theoretical perfect linear match between the values.

# Further discussion

To further understand the implications of the morphological assumptions made when using callipers, we conducted in-silico experiments. In these experiments we showed that changes in the initial conditions give rise to different physiologically-relevant morphologies (see Figure 5 of the main paper).

Single, double and triple peaked morphologies, as well as igloo and birthday cake, are not only relevant to the tumour morphologies observed in standard practice, but also to understanding the mechanisms which generate them. However, we believe that the volcano morphology is unlikely to have arisen from a toroidal initial condition (as modelled here), but rather from the caving in of the tumour due to acute ischeamias which precipitates ulceration and skin rupture. However, for the sake of illustration, the simpler approach adopted here yielded sufficiently accurate results.

We have demonstrated that the shape and location of the distributions of TGI and AUC for the simulated calliper and ground truth differ significantly. The standard deviation of the distributions was larger for SC and there was a downward shift in the size of the effect. This means that the morphological simplification of callipers introduces more variability in the measurements and lower size of the effect. The ratio between these two determines the power of the study, being the theoretical power of callipers lower than that of a perfect surface measurer. There is a morphology-dependent shift in the power of the study, being the igloo-like morphology the most diverging one.

Our sensitivity analysis determines that the height is largely dictated by the proliferation ratio and height bias (by definition), and it is linearly bounded by them. On the other hand, the TGI and AUC depend and are sigmoidally bounded by the length and proportion kill of the treatment. These effects follow from the definition of the problem and indicate that the method is well-posed. Further, AUC and TGI are linearly dependent as is the relationship between AUC for GT and SC. These relationships are systematically biased, where the TGI is lower for the SC. We suspect that in the real scenario, this lower TGI effect can be very accentuated on a case-by-case basis, leading to lowering the power or even invalidating studies. This can cause delays and associated economic losses.

Although these investigations are useful to reproduce different morphologies and understand the shape implication categorically, the span of representative tumour morphologies have not been fully quantitatively investigated. Further, work will be needed to fully characterise the true observed distribution of morphologies and aspect ratios. Anecdotally, we suspect the span of tumour morphologies is more varied than that shown here. However, true distributions of overall tumour morphologies across different tumour models, animal strains, operators, institutions, etc is in the same ball park of the distributions shown above.

1. The First order Moore neighbourhood contains adjacent cells in all directions, including diagonally. [↑](#footnote-ref-1)
